# A Survey of Vehicular VLC Methodologies

**DOI:** 10.3390/s24020598

**Published:** 2024-01-17

**Authors:** Rasha Al Hasnawi, Ion Marghescu

**Affiliations:** Department of Telecommunications, Faculty of Electronics, Telecommunications, and Information Technology, University Politehnica of Bucharest, 061071 Bucharest, Romania; ion.marghescu@upb.ro

**Keywords:** visible light communication (VLC), vehicular VLC, channel modeling, intelligent transportation system (ITS), vehicle-to-vehicle VLC, infrastructure-to-vehicle VLC

## Abstract

Visible Light Communication (VLC) has recently emerged as an alternative to RF-based wireless communications. VLC for vehicles has demonstrated its potential for Intelligent Transportation Systems (ITSs) to exchange information between vehicles and infrastructure to achieve ITS core goals, such as improving road safety, passenger comfort, and traffic flow. This paper seeks to provide a detailed survey of vehicular VLC systems. This paper presents an overview of current developments in vehicular VLC systems and their benefits and limitations for experienced researchers and newcomers.

## 1. Introduction

Vehicular communication is one of the most critical technologies for the development of future intelligent transportation systems (ITSs) [1,2]. Vehicular communication allows cars to exchange information with one another and the infrastructure along the road. For example, infrastructure-to-vehicle (I2V) connections can send extensive, valuable data to link automobiles. Additionally, the data are supplied over an I2V connection (such as the volume of traffic, alternate routes, facilities offered along the road, etc.) [3]. Furthermore, vehicle-to-vehicle (V2V) connections, such as forwarding brake-assist warnings and pre-crash monitoring, are crucial to safety.

Radiofrequency (RF) technology has been commonly used in the majority of the research on vehicular communication [4,5,6]. For instance, the technique of dedicated short-range communication (DSRC) was implemented in [7], which offers a variety of applications, incorporating, among other things, automatic braking warnings and alerts for potential dangers at intersections. Furthermore, vehicular communications based on radio frequency (RF) face some limitations, such as the following: (i) the rate of received packets decreases due to road crowding, especially when there are massive vehicles; (ii) Intelligent Transportation Systems (ITSs) need improved reliability and, in particular, must address several security and safety risks [8,9,10]. Optical Wireless Communication (OWC) is one of the promising technologies for the new generation of wireless networks to overcome most of the problems facing traditional RF wireless communications. Moreover, visible light represents a part of OWC.

**Figure 1:**
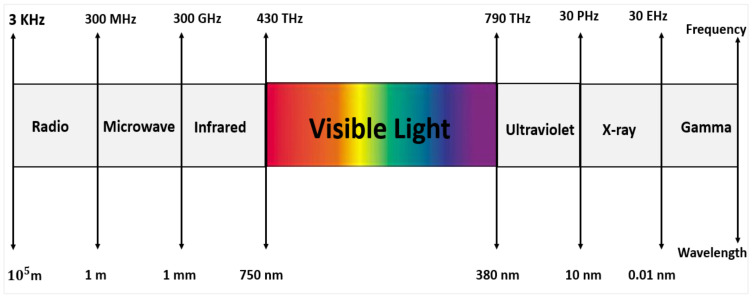
Visible light spectrum region.

Visible Light Communication (VLC) is a new wireless communication method that uses white light-emitting diodes (LEDs) to transmit information and illumination, which is a portion of the electromagnetic wave with a wavelength of 380–750 nm and a frequency range of 430–790 Hz, as shown in Figure 1. VLC technology has unique advantages over RF because its frequency range is safe for the human body and does not interfere with sensitive electronic devices. Moreover, its frequency range is not licensed because no laws restrict it. In addition, it offers free and massive bandwidth, and it is used for both communication and lighting purposes simultaneously. Another key advantage is that it is energy efficient because VLC uses LEDs (presenting small size as well as energy and cost savings) [11,12].

VLC can be used for indoor or outdoor applications, depending on the application environment. Due to the term Li-Fi success [13], indoor VLC has gained more popularity and expansion. Furthermore, outdoor VLC has advanced more slowly due to the more challenging environment and other limitations with regard to mobility, weather, and legislation; it has nonetheless produced significant results. ITS is currently one of VLC’s most potential outdoor applications. Therefore, vehicular networking applications can use the infrastructure for transportation and illumination with LEDs [14,15].

In the context of vehicular technology, all sorts of vehicle-to-everything (V2X), vehicle-to-vehicle (V2V), and vehicle-to-infrastructure (V2I) connections have become suitable to work with VLC due to the widespread use of LEDs in most infrastructure and traffic lights as well as vehicle headlights (HLs) and taillights (TLs) [16,17].

Due to the numerous features described above, VLC technology has greatly interested researchers in both practical and theoretical scenarios, as highlighted by several surveys conducted over the past few years. For example, the authors of [18] presented a comprehensive study on visible light communication, discussing significant challenges and concepts of VLC. Their study focused on the communication architecture of transmitter-receivers, standardization efforts, physical and MAC layers, processing time, and energy efficiency in VLC. In [19], the investigation centered around VLC applications and its historical development. The primary emphasis was on uplink transmission from the user to a VLC access point, aiming to achieve a wide spectrum of visible light and high data rates, while also analyzing the efficiency level and performance of the VLC system. Addressing practical limitations, the review in [20] described channel models, transceivers, computational complexity, efficiency performance, and VLC systems for the physical layer, along with the algorithms used. Additionally, an indoor localization algorithm was proposed, focusing on fingerprints and discussing various aspects of VLC applications, including V2V communications, robotics, healthcare, and national defense. Summarizing the developments related to localization systems using indoor VLC, [21] examined several techniques, such as polarized light, light intensity fingerprinting, shadows, spatial beams, and reflectors, comparing them to other methods. The study concluded that VLC is a viable option for indoor localization, playing an essential role in navigation and intelligent building applications, while offering energy efficiency, low processing time, and minimal computational complexity. Furthermore, based on the techniques presented in the review in [22], VLC can provide seamless, high-capacity broadband connections, potentially reaching data rates in the gigabits-per-second range. In addition, [23] focused specifically on vehicular VLC technologies, with researchers discussing channel characteristics, identifying problems, and addressing the most critical challenges in vehicular communications.

On the other hand, the benefits of using intelligent transportation system applications with the latest VLC technologies were discussed in [24]. The factors that hinder their implementation in transportation infrastructure were discussed as possible suggestions to overcome the challenges. This survey in [25] mainly focused on integrating RF with VLC systems and the associated challenges and benefits. Additionally, the authors discussed the design of VLC systems, including their advantages, performance, limitations, and channel modeling. In [26], a comprehensive study on the theoretical foundations of VLC was introduced. The authors discuss various models for major VLC applications, such as indoor, underwater, outdoor, and underground applications. Finally, the study in [27] discusses machine learning (ML) application scenarios and the architecture of VLC. Many essential methods of VLC are also discussed, such as light modulation and channel modeling. Table 1 summarizes the surveys mentioned above and lists the subjects covered by these surveys.

The concept of vehicular VLC is a crucial enabler for ITSs to improve road safety, traffic flow, and fuel consumption efficiency, facilitating the exchange of critical information between vehicles and infrastructure and having an important impact on the development and progress of transportation systems. Thus, this will lead to a fundamental change from autonomous to cooperative driving, with all the capabilities of the next generation of transportation systems [3,23,28,29].

On the other hand, vehicular VLC systems face several challenges that are fundamentally different from outdoor applications, including the common absence of the line of sight in outdoor applications as a result of reflections from buildings, roads, and streets, in addition to weather conditions (such as rain, snow, and fog) and the effects of artificial lighting and sunlight. Another significant effect is the varying intensity of LED lights, unlike in indoor applications. Realistic channel models are the first step to solving most of these challenges in vehicular VLC systems. Earlier results have focused on indoor channel modeling, which does not apply to vehicular VLC systems with inherently different characteristics. For example, earlier works assumed the ideal Lambertian model for vehicular light sources, which fails to match the illumination characteristics of automotive headlights, taillights, traffic lights, and streetlights with their asymmetrical intensity distributions [30,31,32,33,34,35,36,37,38,39]. The effects of road reflectance, road type, weather conditions, the orientation of the user/vehicle equipment and infrastructure, receiver aperture size, and sunlight might strongly affect the link performance of vehicular VLC systems. Therefore, it is urgently necessary to investigate this research area and its unique uses in traffic safety based on realistic channel models. 

This paper presents an in-depth and comprehensive survey of vehicular VLC systems, covering various critical aspects including a technology overview, system components, signal models, potential applications, environmental challenges, and emerging research areas. It investigates recent advancements in realistic channel models, explores different types of vehicular VLC links, and introduces novel techniques such as reconfigurable intelligent surfaces (RISs), multi-hop relaying, and digital twins. 

The technology overview section provides insights into the fundamental principles, operation, and advantages of VLC technology, along with an examination of essential system components such as light sources, receivers, photodetectors, and modulators. Signal modeling and indoor scenarios are thoroughly explored, with a focus on understanding the main signal models used in VLC and the unique considerations for indoor environments compared to outdoor settings. The potential applications of vehicular VLC are examined, highlighting its transformative impact on the automotive industry and intelligent transportation systems, including lane changing assistance, intersection assistance, platooning, and V2V/V2I communications. Environmental challenges are addressed, investigating factors such as road reflections, interference, and weather conditions, emphasizing the importance of mitigating these challenges for robust communication. 

The survey also discusses emerging research areas, including realistic channel models, different vehicular VLC links, the integration of RISs, and multi-hop relaying, all of which offer promising opportunities to enhance performance, reliability, and scalability. Looking to the future, two key directions are highlighted in this survey. The utilization of digital twin technology is explored, emphasizing its role in optimizing RIS elements and enabling smart, connected, and automated VLC-based vehicles. Additionally, the application of AI and ML algorithms in vehicular VLC systems is discussed, showcasing their potential to optimize resource allocation, enhance security, and enable intelligent decision making. Furthermore, the integration of vehicular VLC with mm Wave networks is proposed as an open research topic, highlighting its potential for achieving continuous connectivity, low latency, and advanced vehicular services. 

The remaining parts of this paper are organized as follows: In Section 2, we explain the basics of VLC technology, explaining the main system components; we also discuss the advantages and challenges of VLC technology and explain the main applications of VLC in indoor scenarios. Section 3 provides an overview of the vehicular VLC system, explaining the motivations for its use in the vehicular field and then reviewing the existing surveys related to technical research works in the literature. Section 4 illustrates several technical challenges in vehicular VLC systems and potential solutions. Section 5 discusses vehicular VLC connections/links and applications. Section 6 provides a detailed discussion of vehicular VLC channel modeling and propagation characteristics, explaining the main differences between indoor and outdoor channel models. Section 7 discusses new research trends, related technologies, and their roles in vehicular VLC systems and gives insights into our future research work. Finally, the paper is concluded in Section 8.

## 2. Visible Light Communication

### 2.1. General VLC Architectures

**Figure 2:**
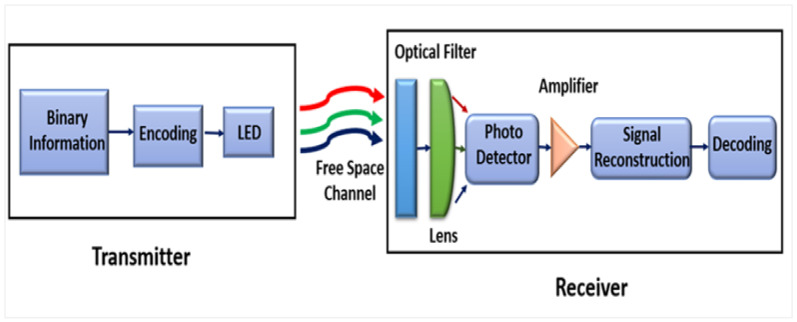
VLC block diagram.

The VLC system consists of three main components: a VLC transmitter, a VLC receiver, and a VLC channel. The essential work of VLC technology is effortless; on the transmitter side, the white light generated by the LEDs is modulated, and then the optical signal is transmitted through the optical wireless channel. The modified light is extracted using a photodetector (PD) on the receiving side. The most popular method for modulating information on the transmitter side and demodulating it on the reception side is known as Intensity Modulation and Direct Detection (IM/DD). A simplified block diagram of the VLC system is shown in Figure 2 [12,18,29].

A. VLC Transmitter

VLC transmitters enable the conversion of data into modulated light emitted by LEDs, allowing transmission through the optical channel medium, as depicted in Figure 2. The transmitter serves a multifunctional purpose by simultaneously emitting both light and data. It generates white light through the LED while modulating the light with the information, ensuring that the transmission of data does not compromise the primary functionality of the transmitter. Additionally, the transmitter must fulfill lighting requirements, maintaining the same light power or supporting dimming capabilities. The modulation applied for dimming should align with the LED lights supported dimming levels, aiming to avoid flickering and ensure a stable light output [40]. There are four types of LEDs utilized in VLC applications, each with unique features suitable for specific use cases. The first type is the phosphor-converter (PC) LED, followed by organic LED (OLED), multi-color lighting RGB LED, and Micro-LED (µ-LED) light. Organic LED lighting is a cost-effective option with limited bandwidth (around one megahertz [41,42]), while µ-LEDs offer a significantly larger bandwidth (approximately three hundred megahertz). RGB and PC LEDs are more commonly used than single-channel LEDs, with RGB LEDs providing a high output, equivalent to three times the modulated data output due to their three independent color channels. However, white PC LEDs are frequently preferred, as they are easier to implement and more cost-effective compared to RGB LEDs [43]. To achieve data modulation using the light emitted by LEDs and control the current flowing through them, a driver circuit is employed in conjunction with the LED light source [44].

B. VLC Receiver

The receiver device is one of the essential elements in VLCs, where the received light signal is detected using photodiode detection devices (PDs), which work to extract the electrical signal from the received light signal. These devices are either stand-alone or may be in the form of an image sensor (or the so-called camera sensor), which consists of several diodes arranged in an array. Moreover, image sensors are also used in mobile devices (smartphones). However, the throughput rate realized is limited (kilobits per second) [12]. In the meantime, photodiode (non-imaging) devices have a higher data rate when receiving the signal (up to hundreds of megabits per second), so the PDs used in VLCs vary widely. Furthermore, four types of photodiodes are usually used in VLC applications. The first type is the Avalanche photodiode (APD), the second type is intrinsic positive–negative (PIN), the third type is the metal semiconductor metal PD, and the final is the photo-conductor. Therefore, both PIN and APD photodiodes are the most popular and widely used because of the features that enable them to implement the requirements of VLC systems [45]. All types of photodiodes used in VLCs work to obtain the electrical current from the received light. Then, the received electric current is amplified before it reaches the equalizer. On the other hand, the optical condenser can be used in the future by focusing the received rays in the photodiode, as seen in Figure 2. At the same time, the VLC receiver contains an optical filter that removes unwanted noise to enhance the efficiency of the VLC system [43].

C. VLC Channel

In a VLC system, the connection between the transmitting device and the receiving device occurs through the optical carrier medium in free space. It is important to note that light primarily functions as an electromagnetic wave, and its strength diminishes with the square of the distance, similar to other wireless communication systems. When designing VLC applications, several crucial factors need to be taken into account, particularly the disparities between indoor and outdoor environments, with interferences having a more pronounced impact on indoor applications [46,47]. Interferences in indoor settings can be caused by reflections from walls or objects within the space, leading to symbol interference, or by neighboring users, resulting in co-channel interference. On the other hand, outdoor applications are influenced by various surrounding environmental factors, which necessitate consideration [29]. These factors include the noise generated by sunlight and artificial light sources, as well as attenuation caused by different weather conditions in the atmosphere, user movement or traffic, and the density of users.

**Figure 3:**
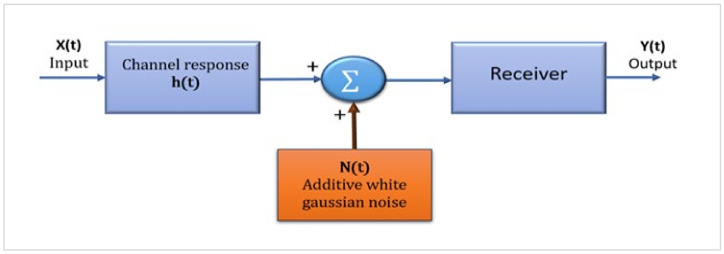
VLC channel model structure.

On the other hand, the light intensity profile is an essential factor through which the channel is described in VLC systems because it mainly affects the interactions of environmental elements with the emitted rays during their path until they reach the PD. The ideal Lambertian model usually describes indoor lighting, because it has uniform radiation. However, this does not apply to all LED light sources with wildly irregular light patterns (asymmetric patterns), which are usually the patterns in outdoor lights. However, photodiode surface areas play an essential role in the VLC channel because they mainly affect the total number of captured beams and thus affect the real power collected. Therefore, the channel modeling method for VLC systems is essential to evaluate the system’s performance. The modeling method is applied on the basis that the response is a non-negative pulse h(t) > 0 and at a Linear Time-Invariant (LTI), as shown in Figure 3 and expressed as follows [26]:

Y(t) = RX(t) × h(t) + N(t) (1)

where Y(t) represents the electrical signal that has been received, R represents the responsivity of a photodiode, X(t) denotes the input light power, h(t) represents the channel impulse response, (*) represents convolution, and N(t) indicates the noise of the receiver (additive white Gaussian noise (AWGN)).

Moreover, light rays can be refracted or reflected through the channel or scattered in sub-rays. Therefore, the VLC signal arriving at the receiving device is the sum of two essential components: the first component is a line-of-sight (LoS), where the light signal reaches the receiver directly, and the second component is non-LoS (NLoS), where the light signal reaches the receiver after reflections and scattering due to obstacles [26]. 

### 2.2. Purposes of the Visible Light Communication

1. VLC Advantages

VLC is considered among the essential enabling technologies for the next generation of wireless communications due to its unique features and advantages. VLC technology has all the benefits of the visible light spectrum, including large bandwidth, being license-free, and being safe for the human body and electronic devices. Additionally, VLC can handle data transmission and lighting simultaneously. It is also considered more reliable than RF, so VLC systems solve most of the difficulties encountered in RF connections. Apart from all these advantages, VLC is a low-cost solution that is also easy to use. The following are the main benefits of VLC.

A. Large Unlicensed Bandwidth

Radiofrequency wireless systems typically cover a range between 3 kHz and 300 GHz. Every wireless network has its available bandwidth, to prevent interference. Due to the limited availability of licensed spectra, obtaining national authorization and approval is extremely important. Furthermore, as wireless technology usage and requirements expand, such authorized and prescribed elements have the unintended consequence of reducing the radio spectrum. Thus, there is a need to create alternative energies with maximum throughput and spectrum reduction. The visible light spectrum, “Equivalent to the 380 to 780-nanometer frequency band “is 10,000, which is much more significant than the range of radio signals [11,12]. Moreover, VLC is not subject to spectrum control or license compared to radio frequency communication. VLC offers ample unrestricted bandwidth resources for transmitting data, providing a possible response to the radio frequency spectrum constraints in wireless technology.

B. Secure for Human Health

Radio frequency risks have received a lot of attention recently. Extended exposure to intense electromagnetic waves may negatively affect how well the human body functions. As a result, wireless communications must generally limit their transmitting power. This seriously influences the performance of the system. The VLC illumination is provided by LEDs that emit white light, which is entirely harmless to humans and similar to normal light. When health factors are considered, VLC communication becomes the most popular option [11,48].

C. Security and Interference

Indoor communications within a building or room are susceptible to eavesdropping due to radio waves’ ability to travel through walls. Additionally, the usage of radio waves is severely constrained due to reciprocal interference with electrical equipment. The security of VLC is substantially strengthened because light may be firmly limited to a specified area, enclosed by impenetrable barriers. According to a recent study, it is possible to eavesdrop on VLC because light signals can seep through gaps between the ground and doors, partially obscured windows and keyholes [11,49]. Therefore, VLC is much more reliable than conventional RF connections. VLC produces no potential health risks and cannot interfere with or impede radio waves. It is an excellent method to employ in delicate electrical equipment, including in hospitals and commercial and aerospace industries [50].

D. Energy-efficient and inexpensive

VLC systems remain substantially less expensive compared with conventional radio communication. According to research [51], a Bluetooth piece with a maximum data transfer rate of one-megabyte costs at least USD 5.00, while a VLC with a transfer rate of roughly four megabytes costs only about USD 1.00 per piece. Furthermore, due to the excellent energy consumption of LEDs, the VLC-based LED is emerging as the best friendly connection method solution. LEDs utilize less energy [14] and produce less heat than traditional lighting systems. LED lighting consumes 1/4 and 1/10 the power of conventional and fluorescent illumination.

2. VLC Challenges

Despite the many advantages that VLC systems possess, they contain some defects. Most defects date back to the beginning of VLC technology and may be bypassed when the technology is fully developed. Another reason is the way light is used and its characteristics. It is not easy to eliminate these, but their impact can be mitigated, and communication can be adapted to the circumstances.

- Line-of-Sight (LoS) Blockage

Line-of-sight (LoS) connection is often considered necessary for VLC systems, as it raises system efficiency and reduces multi-path distortion. The required LoS state can be considered beneficial in some cases. On the other hand, other applications see this as a significant drawback because the requirement for LoS restricts motion. In some cases, visible light is a considerable drawback when the barrier prevents communication from progressing when no other path is accessible [43,52,53]. Furthermore, communication links can be provided while using intelligent reflecting surface (RIS) technology, digital twin technology [54], and multi-hop relaying techniques [55].

- Limited Transmission Range

VLC technology has a greater bandwidth than radio frequency. However, the transmission range of VLC is smaller than RF. The transmission range is increased by first improving the characteristics of VLC transmitters and receivers; secondly, by increasing the transmitting power by using a more focused and directed light beam; and thirdly, by expanding the communication bandwidth on the emitter side or increasing the field of view on the receiver side; however, this can increase interference. In all of the above cases, VLC communication is much shorter than radio frequency communication [56,57,58,59]. On the other hand, the communication range is improved by using several techniques that increase the signal-to-noise ratio (SNR) by using an optical lens or a narrow FoV receiver; also, by using different filtering techniques or through the multi-hop connection, the connection capacity of VLC systems can be significantly increased.

- Interference and Noise

VLC is affected by light from other sources, including artificial sources such as fluorescent and incandescent light; these usually produce low-frequency noise, which is eliminated using high-frequency filters. On the other hand, VLC systems are affected by sunlight, where sunlight creates unmodulated light that is a direct electrical current having a strong effect on the receiver [19,60]. The received signal is improved, and noise is removed using capacitive DC filters. Still, high-density optical noise hinders communication and saturates the receiver. Moreover, the receiver’s low field of view and optical and electrical filters can eliminate unwanted noise. Although these technologies mitigate the impact of interference, the VLC connection remains affected by high noise levels.

3. VLC Application

VLC technology has gained popularity in various applications due to its appealing features, including high bandwidth, low power consumption, unlicensed channels, and absence of health risks. It can serve as a standalone solution or complement radio frequency communication to achieve high-speed and efficient data transmission. VLC applications can be classified into three main categories based on their respective areas of application: indoor, outdoor, and underwater. 

Indoor VLC has found widespread use in numerous applications, some of which are:

- Li-Fi

One prominent application that has emerged for VLC’s high-speed capabilities is Li-Fi, also known as “optical Wi-Fi” or “light fidelity.” The term Li-Fi was coined by Professor Haas in 2011, leading to the establishment of the Li-Fi consortium to harness the technology’s rapid development and immense potential [11,13]. Li-Fi functions as a bidirectional system similar to conventional Wi-Fi, but with the advantage of leveraging VLC’s extensive bandwidth. Through ceiling lamps, Li-Fi enables high-speed internet connections within a limited range, typically within a few meters between the ceiling and the office floor. Li-Fi technology can provide multiple Gbps connections. The fundamental principle behind Li-Fi is the conversion of internet data into light signals for transmission, which are then received by the recipient device (e.g., mobile terminal) and converted back into electrical signals and digital data.

- Localization

VLC-based localization is particularly suitable for indoor environments where GPS systems cannot operate effectively. The presence of LED lighting ubiquitously in indoor spaces, including subways, buildings, hospitals, and airports, presents unique opportunities for VLC-based indoor localization. By utilizing visible light identifiers and extending the system when identifier coordinates are available at the transmitter, VLC-based localization can accurately determine the position of objects or individuals within a room. Different methods can be employed for localization, such as optical tracking and imaging, as well as triangulation/trilateration. A key requirement for all localization applications is the ability of the receiver device to capture signals transmitted from each lighting unit separately [11,58,61].

- Smart Homes

Smart homes facilitate seamless communication between residents, government entities, infrastructure, the economy, and the environment [58,59]. While most components of a smart city are already in place, achieving integration of all these components necessitates high-speed and reliable wireless communication. VLC technology can leverage existing indoor lighting sources to provide indoor hotspots capable of delivering extremely high data rates. This enables essential services and sustainable networking capabilities within smart homes while preserving the valuable radio frequency spectrum used by smartphones. To ensure smooth integration of VLC technology in smart home applications, a three-layer VLC-based communication structure has been proposed [62]. In this architecture, VLC is used in the first layer to provide users with access and situational awareness. The second layer facilitates communication between different LEDs and sub-gateways. The final layer enables optical communication between the service gateway and multiple sub-gateways. Based on this architecture, various applications have been developed, including event monitoring, intelligent communication, and detection and tracking.

## 3. Vehicular VLC Overview

### 3.1. General Context

Congestion in emerging countries and worldwide has become of great importance due to the significant problems it causes, such as difficulty in transportation, high fuel prices, and environmental pollution issues. Moreover, the increase in the number of vehicles often causes accidents, according to [63], as human errors are responsible for about three-quarters of car accidents; therefore, if there are warning devices on the road, the probability of accidents will decrease significantly. Intelligent Transportation Systems (ITSs) have effectively helped address most of the abovementioned difficulties. The importance of high-speed wireless communication has increased, enabling development of these systems, which have many essential applications, including pre-collision warning and emergency braking, sensing or warning when changing lanes or before a collision, and alerts at road curves, in addition to assistance in turning and stopping movement and when violating a traffic signal [64]. Conversely, data can be exchanged between vehicles, including speed, location, engine status, acceleration, etc. Intelligent transportation systems (ITSs) have been the subject of intense research efforts over the past decades, as these systems will be essential parts of smart cities in the future [29,65]. ITSs enable the process of exchanging information between infrastructure and vehicles. The main objective of ITSs is to ensure safety, reduce traffic, and reassure passengers.

Additionally, major companies like Google, Tesla, and Uber have assessed fully autonomous vehicles as an essential standard for ITSs. Moreover, necessary enabling technologies have emerged that have helped create advanced transportation solutions. Among these solutions are vehicle-to-vehicle (V2V) and pedestrian-to-vehicle (P2V) communications, also called communications between vehicles and everything (V2X), and vehicle-to-infrastructure (V2I) communications [4]. 

The practical application of intelligent transportation systems requires flexible, highly reliable, and scalable solutions. Furthermore, due to the expansion of standardization activities and research efforts on vehicle communications to everything, with the majority of the work depending on RF technology, through which short-range communications (DSRC) have been used by commercial vehicle manufacturers [66], as well as due to cellular-V2X communications for the fifth generation (C-V2X), which is now being built [67], radio communications are now suitable for ITSs because vehicle communications to everything in the market have become shallow. However, the interference levels may become significant in the future, especially when conditions are congested. In addition to the restricted bandwidth, this causes a lot of delays and a low packet rate due to congestion, which affects communication channels. Therefore, visible light communication (VLC) technology has emerged as an alternative candidate or a complementary solution to address most of these problems [29,68]. 

Additionally, there is widespread use of lights for traffic infrastructure, especially light-emitting diodes (LEDs), as well as headlights (HLs) and taillights (TLs) in vehicles; these lights have some of the features that make them an alternative solution to traditional halogen bulbs. Intelligent transmission systems make VLC a possible option. The most prominent features are brighter lighting levels and a high-rapidity switching function, which ensure longer lifetimes and minimize heat dissipation [16]. Over the last decade, research into VLC systems for indoor applications has received significant attention [12,14]. Nevertheless, their uses in outdoor contexts, like vehicle communications, are still relatively new and require further research [15]. Vehicular VLC seeks to achieve concurrent illumination and information data transfer by equipping vehicles with a light-emitting diode (LED) because of its resistance to RF interference, jamming, and spoofing, as well as the availability of its massive spectrum without the necessity for licensing and the line-of-sight (LoS) network connectivity it offers; it is also considered a safe and alternative technology when compared to RF communications. Sometimes, it is regarded as a it to support many applications, including platoon, safety, and critical applications, which require adherence to many demanding standards. Besides LoS links in VLC systems, line-of-sight links are usually unavailable in vehicular VLC applications due to restrictions from the ground, buildings, cars, and other objects’ environments. To create the line-of-sight (LoS) links for VLC systems, some studies have suggested using intelligent reflective surface techniques (IRSS) and multi-hops between vehicles and users, in addition to digital twin technology (DT), which was recently used to provide effective improvement and to meet various requirements for emerging applications. It also helps in improving the phase, capacity, and frequency transformations of IRS elements installed in the infrastructure of roads, cars, bus stations, and buildings. Moreover, weather conditions like rain, fog, ice, and snow affect the strength of the LoS links, and these effects can also cause reflectivity [23,69].

**Figure 4:**
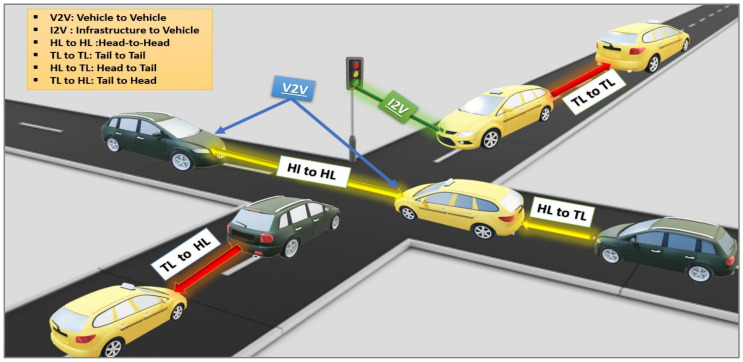
Connections between vehicles that utilize vehicular VLC, comprising tail to head, head to tail, head-to-head, tail to tail, and infrastructure to vehicle (I2V).

The typical flow of traffic that vehicular VLC can accommodate is seen in Figure 4, and vehicles can communicate with cars with several other automobiles close to them. As a result, they can establish tail-to-head and head-to-tail connections while they are moving, along with head-to-head linkages while meeting one another at intersections. There is also the possibility of a tail-to-tail link; however, this type of connection is not very prevalent in normal driving conditions. Figure 4 shows the I2V relationship, which includes infrastructure elements using LEDs, including roadside streetlights, traffic lights, and signs, where digital information is sent to and from vehicles via a VLC connection, and necessary and visible information is sent to drivers [23,70,71].

### 3.2. Reviews on Vehicular VLC

There is already a substantial amount of research to explore the performance of V2V and I2V VLC lines, and this research is expected to continue to expand.

A. Vehicle-to-vehicle reviews

The authors in [36] presented a channel modeling study on a V2V link and used an asymmetrical high-beam headlight at the transmitter side. The study focuses on developing a path loss model under different weather conditions to determine a maximum distance of about 72 m in clear weather conditions and 26 m in fog conditions. Moreover, road reflection models and the intensity distributions of headlights were also considered. On the other hand, the proposed closed-form formulation for the path loss is only relevant for a minimum distance that does not exceed 20 m. Furthermore, a non-constant random geometry-based model (GBSM) is suggested for V2V VLC channels. According to the results, the amount of power loss varies greatly depending on the distance, the height of Rx, and the pattern of the optical source [72]. In order to support longer communication distances, the authors in [39] presented a negative exponential form, which produced a good fit with the findings of the ray tracing simulation under various atmospheric circumstances. The authors calculated the shortest distance to travel while meeting the BER requirement. According to the findings, it was possible to travel a maximum distance of approximately 72 m when the weather was clear, but this distance was only about 26 m when fog was present.

The implementation of a twin photodetector and an angle diversity receiver contributed to performance enhancements [56]. Moreover, there is a significant variance in the ray tracing scenario for path loss calculation when compared to the Lambert model, as shown in [73], where the authors present a non-sequential ray tracing simulation and focus on the radiation patterns of different taillights to investigate the performance of V VLC, to examine the effect of neighboring vehicles when delays spread to various scenarios. Additionally, the effect of neighboring vehicles when delays spread to various scenarios. A precise channel characterization of VLC-based V2I systems is provided. [74]. This type of model is capable of being practically applied to TLs manufactured by a variety of different companies. As a result, it is simple enough to be utilized in estimating the network performance of V2V systems. According to this concept, the maximum transmission distance that could support reliable communications was as follows: contact was made possible with a bandwidth of 100 kHz and a range of up to 66 m thanks to this technology. The effect of fog on vehicle-to-vehicle (V2V) connections was studied in [75]. The authors presented an experimental method on a VLC link based on a camera at the receiver side using natural light in the transmitter, considering the distance and visibility levels between vehicles. Moreover, the results are shown when using the signal modulation index decreases from 1 to 0.75 and 0.5: the connection error rate is up to 20 m, and it deteriorates further at the 0 index to reach about 10 m.

Furthermore, for achieving omnidirectional coverage for V2V under various practical scenarios of imperfect alignment and lane changes, and presenting the total contribution of received optical power considering all PDs, [37] proposed a VLC-based channel modeling method for a V2V link between a source vehicle equipped with a transmitter-side high-beam, where four photodiodes support the car on the destination side. The first two parking PDs are in the car’s trunk, directly below the taillights. Moreover, another one is located on the left part of the vehicle, and the final one can be found in the right position. Another work that investigates the maximum achievable distance with various parameters is [76], which introduced the performance of vehicular VLC systems based on non-sequential ray tracing to obtain the channel impulse responses under different weather states. Moreover, the closed-form path loss expression is proposed for both geometrical and attenuation loss. It considers lateral shift; link distance; clear, rainy, and foggy weather types; receiver aperture diameter and transmitter beam divergence angle; and asymmetrical light. In [69], the effect of shadowing and weather disturbances on V2V VLC communication performance was studied, as shading causes blockage of the line-of-sight path. Furthermore, the system was analyzed using diversity–multiplexing tradeoff, as it provides an optimal tradeoff between multiplex gain and diversity gain. The authors in discussed the effect of external propagation characteristics on vehicle-to-vehicle (V2V) communications for both line-of-sight (LoS) and non-line-of-sight (NLoS) models, where the path loss was about ≈6 dB. The received power was obtained as 7 dB in [77] by using a non-sequential ray tracing approach to study the performance of the V2V VLC system, where the effect of different types of lenses in a receiver was studied, including the combined lens, Aspherical lens, and Fresnel lens, in addition to the impact of bandwidth and receiver diameters, taking into account the asymmetric headlights of the vehicle. In [78], multiple input and output (MIMO) systems were presented with the vehicle-to-vehicle VLC system, where the horizontal and vertical lateral shift was discussed, and the effect of weather conditions, artificial light sources, and optical channel modulation orders between vehicles were studied by examining the performance of SNR and BER, as MIMO technology can significantly enhance system performance.

Most of the previous literature focused on studying individual technology. At the same time, in [79], millimeter wave (MMW) and vehicular visible light communications were used. A comprehensive comparison between MMW and VLC channels was presented, and the channel characteristics were investigated between two vehicles with side shift in different paths in the same direction, providing analysis of the density effect of neighboring vehicles.

B. Infrastructure-to-vehicle reviews

The authors in [74] conducted an analysis of the performance parameters of a Vehicle-to-Infrastructure (V2I) connection using Visible Light Communication (VLC) in a two-road scenario, where automotive headlights served as transmitters to connect with traffic light poles. They established that the road infrastructure met the necessary criteria, with the traffic light poles featuring colored lighting (yellow, green, and red) and three nearby photodiodes. Realistic Channel Impulse Response (CIR) reports were generated using ray tracing, demonstrating the impact of LED front-end characteristics on channel performance and the influence of optical CIRs. The study also explored the effects of lateral shifting on V2I communication by initiating measurements from various positions while the vehicle was in motion. Based on these parameters, calculations were performed to determine the maximum achievable data rates in each V2I scenario.

In another work, [35], coordinated multipoint transmission (CoMP) was employed to establish a dynamic handover method. The proposed method considered variations in received power levels and recalculated delivery aspects and start-up time values without explicit information about the vehicle’s speed. Results indicated that the proposed method outperformed conventional CoMP in terms of consistent signal quality during handovers, irrespective of vehicle speed. The study also examined how vehicle speed affected the received signal-to-noise ratio. Additionally, to achieve low latency in VLC system communication for traffic signals, the authors employed empirical characterization and analysis of optical performance [80]. They used a typical optical signal, considering the effect of optical diodes and custom amplified optical capacitors, to transmit digital information to the receiving stage. The packet error rate was calculated in terms of the effective field of view (EFOV). System performance measurements were taken at varying distances between the receiver and transmitter, using a wide range of optical capacitors from 3 to 50 m. The study showcased different responses based on the angle, with lens cases exhibiting an EFOV greater than 150 degrees being unsuitable for distances beyond 20 m. The findings demonstrated a range of responses depending on the angle due to the measuring connection’s placement. Moreover, the lenses were observed to significantly improve the packet error rate from 105 up to 50 m while maintaining an EFOV of more than 10 degrees.

Another study utilized non-sequential ray tracing to analyze and describe the effectiveness of Infrastructure-to-Vehicle (I2V) connections using street lighting [36]. The authors provided closed-form expressions for path loss terms, probability distribution functions (PDFs), and connection parameters. They considered parameters such as column height, receiver aperture size, lateral displacement, and distance between traffic illumination poles and assessed their impact on the system and bit error rate. The proposed path expression was compared with Monte Carlo simulations, with the aim of achieving higher accuracy and complexity compared to the conventional Lambertian pattern model. The Lambert pattern exhibited an error of 25%, while the suggested polynomial models, with 12 coefficients, achieved an error of about 8%. In [81], a VLC channel was specifically designed for I2V communications with traffic lights in a real urban road scenario. Three models were proposed to describe the connections between traffic lights and vehicles, considering the limitations of the Lambert model. The accuracy and complexity of the proposed models were compared to the Lambert model, revealing a reduction in error to about 8% with 12 coefficients, while the Lambert pattern model exhibited an error of 25%. Additionally, [82] studied the bit error rate (BER) in the infrastructure-to-Vehicle (I2V) system, considering the effects of both lateral and longitudinal shifts, as well as modeling the channel using the non-orthogonal multiple access method (NOMA).

In summary, the above review indicates a pressing need to improve the overall channel model for VLC in vehicles, while considering factors such as weather conditions (clear, rainy, and foggy), the asymmetric beam pattern of headlights, lateral shifting between vehicles, the effects of road reflections, receiver aperture diameter, and transmitter beam divergence angle. Table 2 summarizes the reviewed methodologies for channel modeling and the light sources of VLC utilized in establishing V2V and V2I/I2V links.

C. Prototype scenarios reviews

There are some shortcomings in the works that support real-world scenarios because most of the results rely on experiments and simulations. In this section, we summarize the most important works dealing with the practical aspects of vehicular VLC communications.

The authors in [8] studied the first realistic driving test of the V2V VLC system, where a working range of up to 45 m was achieved. The optical system technique was used in the receiver device, and the OFDM technique was used to send symbols to the transmitter device. Moreover, the angle of incidence and distance significantly impact energy. They were received, with road environments and reflections of neighboring vehicles having little effect on performance. The study also showed that the factor that leads to beam drops is when the LoS paths between the taillights and the receiver are blocked; this provides justification for placing the receiver in the middle, to achieve the LoS paths. LED signs also generate interference. This results in a transmission disruption that encryption techniques can handle. Furthermore, in [84], a system prototype proposed for hybrid communications was introduced by integrating RF radio communications technologies with VLC in vehicles to achieve communication continuity over longer distances. This is because visible light is suitable for V2V communications only within short distances. Therefore, RF communications complement the system, covering all areas and providing in-network information about road infrastructure, spaces, sensor analysis, and traffic.

In [85], a commercial traffic signal transmitter was used to design and test a prototype for I2V VLC communications, where 200 Fresnel lenses were integrated into a photodetector at a receiver, based on an open-source microcontroller platform (Arduino Due) for decoding. The performance of the encoder was also evaluated. PER checked system performance and achieved error-free range coverage (PER ≥ 10^−5^) from 18 to 50 m with lower latency, making it suitable for 5G and cellular-V2X integration. The authors in [81] presented an analysis of the optical and communication performance of I2V VLC systems, where a typical traffic signal was used to transmit information to the receiver in a realistic implementation. The receiver was used on an amplified photodiode and an optical condenser, in addition to receivers in the no-lens case, and the results were shown. The use of different lens combinations is essential in the design of I2V VLC systems because they are realistically effective in ITS applications. The system was tested and compared for distances between 3 and 50 m, as a no-lens case does not achieve communication over distances greater than 20 m, even though it has a field of view exceeding 150 degrees. In [86], the prototype of the V2V VLC was tested under exposure to fluorescent lamps at night and in daylight conditions. The study focused on two degrading factors: the effect of vehicle misalignment and the maximum communication distance. A communication range of 75 m was achieved by relying on the vehicle’s rear lights; thus, a narrow field of view and optical filters can improve the receiver device. However, evaluating the VLC system in real driving situations as compared with experiments remains relatively complex with regard to repeatability of measurement and communication safety and thus requires more effort and testing. In [87], the focus was on using the Angle Diversity Receiver (ADR) for vehicular VLC systems to enable multi-directional signal reception. Different scenarios for movement conditions were studied, such as left turn, U-turn, and right turn. Moreover, software-defined radio platforms were introduced to obtain preliminary results for experimental verification, where a packet delivery rate of 99% was achieved.

### 3.3. Vehicular VLC Architecture

**Figure 5:**
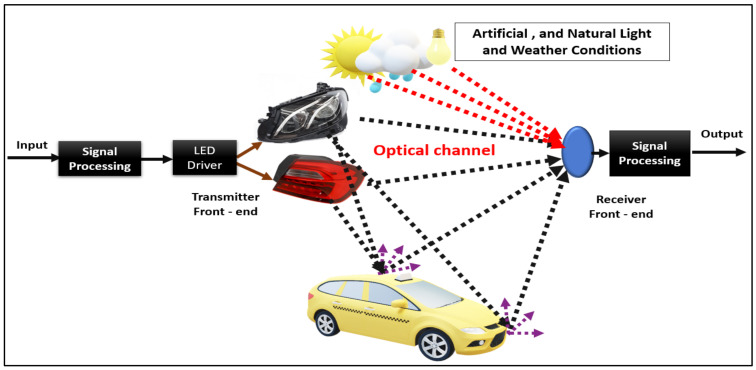
The general structure of the V-VLC system. The figure, on the left, shows the transmitter device, including signal processing (encoding, modulation), LED driver, and transmitter front end. The transmitter and receiver are linked by a channel affected by interference from weather effects, reflections, and natural and artificial light sources. The right side shows the receiving device, including the receiver front end and signal processing (demodulation and decoding).

Figure 5 shows the basic architecture of the vehicular VLC system. The vehicular VLC system contains the transmitter side of the signal processor (modulator) and the front end of the transmitter (e.g., headlight and taillight LEDs). On the receiver side, it consists of optical elements (which can optionally be used in the receiver) and signal processing (a device attached to the receiver that works on decoding and demodulation), and finally, the optical channel, which is the actual distance between the transmitter and the receiver. The LED driving component merges the modulated signal with the bias current required to power high-power LEDs [14] instead of the low-power LEDs used in indoor VLC systems. This manner modulates the transmitter front-end information on the light intensity [88]. Therefore, the design of the driving circuit for VLC systems is essential, as it must provide a correct bias so that the LEDs are lit at optimal operating points and ensure that there is no signal cut-off before the modulation process, because the low operating end of the LED leads to the component may interrupt the negative parts of the signal and furthermore may exceed the high operating point of the LEDs of the linear region. This leads to distortions in the movement or may damage the LED, as shown in [89,90], where some applications are presented using an open-circuit driving source. The light emitted by the LEDs interacts with the optical elements inside the lighting unit, which control the shape of the beam through automotive regulations [23]. The resulting signal depends on the optical factors, which have a substantial effect. For example, lenses are used, through which the light is focused on a specific point in the distance; using different LED lamps, the matrix lights work on the spatial separation concerning the multiple transmissions of that point in different (but overlapping) directions [91]. Moreover, when the light signal propagates in the optical channel, it will undergo different propagation phenomena. The optical signal may weaken due to path loss. Additionally, outdoor channel disturbances cause an increase in its degradation. If not attenuated by the receiver sensitivity threshold, the signal reaches the receiver directly through a link or reflection.

Optical filters can be used on the receiver side, placed in the receiver front end, which in turn helps improve the system and thus increases the signal-to-noise (SNR) because it filters unwanted optical channels (which contain noise). In addition, lenses (optical elements) collect light and focus on the receiver aperture. Then, the signal is amplified through optical pre-amplification, and thus the strength of the received signal is improved [8,17,33,77,92]. A camera image sensor or PD is used at the receiver side to convert optical signals into electric currents. The generated photocurrent is converted into voltage using a transversal circuit [93]. For camera image sensors, the generated photocurrent is converted into voltage via a reading circuit [94]. The signal processing block is the last step in the vehicular VLC system in the receiver. Moreover, receivers have fundamentally different architectures in image sensor-based and PD-based sensors, and the signal processing methods and steps also differ; for example, VLC-based systems do not require PDs to refer to image processing as in an image sensor. The analog current signal during signal processing is converted into a digital signal again, and then the information bits are decoded.

## 4. Technical Challenges in Vehicular VLC and Solutions

Despite the numerous features provided by vehicular VLC communications, some challenges remain due to the use of light as a transmission medium. In this section, we will discuss the most critical difficulties and challenges affecting vehicular VLC communication.

### 4.1. Impact of Reflection

**Figure 6:**
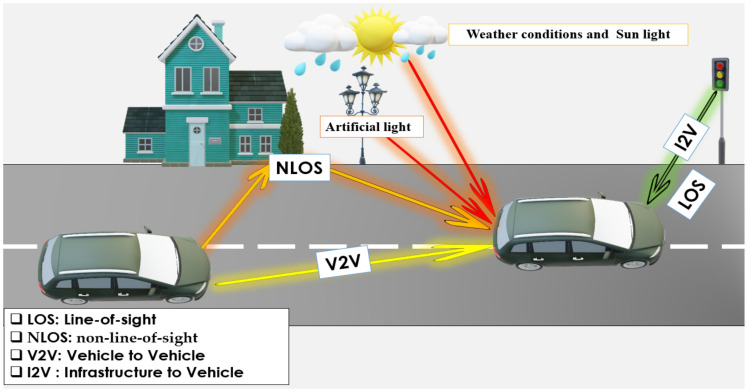
Impact of light sources, reflections, and weather in vehicular VLC.

Line-of-sight (LoS) communication is essential in vehicular VLC. However, its availability is a critical challenge in outdoor applications due to reflections from buildings, vehicles, land, and other objects, which form non-line-of-sight (NLoS) links. Due to these NLoS links, the reliability of the connection cannot be guaranteed, because the signal strength in the receiver device depends mainly on the reflection properties [17,95,96]. Moreover, climatic conditions such as snow, ice, and rain affect the strength of reflections, affecting the road surface and thus influencing the reflectivity and connection strength of NLoS [95,96]. In some instances, the road may be under highly reflective conditions. These reflections from the ground surface are much more substantial due to the angle of reflection, so the wet road is emulated by using a shiny linoleum inner sidewalk. Within certain distances of both the receiver and sender side, the non-line-of-sight components have the potential to have signal strengths comparable to those of the line-of-sight parts. Figure 6 shows the impact of reflection (LoS and NLoS).

The characteristics of the vehicular VLC propagation channel are primarily affected by optical signal distortion caused by reflection, dispersion, and attenuation. These factors significantly impact the performance of the vehicular VLC system, leading to the emergence of research focusing on modeling the vehicular VLC channel under LoS transmission assumptions. In [76], a ray tracing simulation method is proposed to develop a headlight LoS path loss model. Similarly, ref. [73] presents a taillight loss model. Additionally, ref. [30] introduces a path loss model that considers asymmetric lighting conditions. However, there are limited studies addressing the NLoS vehicular VLC channel. The effects of reflections on road surfaces are explored in [96,97], emphasizing their impact on vehicular VLC performance. Furthermore, ref. [95] investigates specular reflections on the road surface and their detrimental effects. Another study, ref. [98], focuses on the reflection from surrounding vehicles. Table 3 provides an overview of the studies discussing the influence of LoS, NLoS, and reflections on vehicular VLC.

### 4.2. Sun and Artificial Light Sources

Another critical challenge is the interference from artificial and natural light sources, which negatively affects the performance of vehicular VLC. This includes the ambient light from artificial light sources (such as fluorescent and incandescent lamps used for roads, traffic lights, infrastructure, and billboards) or natural light sources such as sunlight. In addition, the connection is hampered if there is a significant amount of radiation, defined as a situation where the maximum intensity of the light, both natural and artificial, is greater than the desired optical signal power. It is possible that the background radiation could be too intense in some scenarios (such as being in direct sunlight), leading to the receiver being entirely saturated. The electrical signal created by the photodiode’s output is a direct current (DC), because the light from natural sources such as skylights and sunlight cannot be modulated. The filter is often used on the receiving side, where it works on a low pass to reduce the effect of DC because it is utilized to connect with higher frequencies [23,99]. The impact of light from the sun and artificial sources on vehicular VLC is depicted in Figure 6. Although the background radiation level causes noise, also known as shot noise, the shot noise is generated due to fluctuation in the photons arriving randomly at the PD [17]. The shot noise comes from solar radiation. In contrast, the variation in the shot noise is the result of artificial sources, which may cause significant communication problems during the day; thus, the noise generated is considered a negative factor for the performance of vehicle systems as, especially during sunset and sunrise, different angles of the sun can cause the deterioration and saturation of the photodiode [17,23]. The sufficient bandwidth of the LEDs is likewise diminished when the weather is sunny. Approaches based on optics can also be used to deal with more problems of ambient light and generated disturbance noise. Methods concerning this have already been addressed. Utilizing highly directional receivers that can differentiate the wanted signal from the noise source is one potential approach to resolve the issue [23,100]. Methods based on optics can also be used to deal with more problems of ambient light and generated disturbance noise. One approach that might be taken to resolve the issue is utilizing highly directional receivers that can separate the wanted signal from the noise source [71].

On the other hand, this may lower the system’s tolerance for movement because it will have a narrower field of view (FOV) at the receiver. Additionally, the optical filtering of specific wavelengths, such as the blue filtering of light, has been proposed [101]. In contrast, when utilizing this method, in addition to the noise, significant signal strength may be lost on the wavelengths that are being blocked. The image sensor receivers are of broad importance in terms of receivers, especially when it comes to neighboring or surrounding noise, because they work to separate the source of the received signal from the noise generated, using the method of isolating pixels that include noise; this technique is essential to solve the background noise problem [36,102].

### 4.3. Weather Conditions

The performance and reliability of VLC vehicle channel connections are significantly affected by challenging conditions and weather fluctuations, such as snow, fog, and rain. Figure 6 illustrates the impact of rain on V2V connections. When light encounters particles in the atmosphere, including diffraction, scattering, and absorption, it leads to the attenuation and dispersion of transmitted signal beams. These weather factors and fluctuations consist of larger particles that have a notable effect on the system’s performance, hindering the connectivity of vehicles through VLC. The implications mentioned above become even more severe in unfavorable weather circumstances. Consequently, the range and reliability of the vehicular VLC system are negatively impacted. Initial studies have addressed various weather phenomena to understand their influence on the performance of vehicular VLC. These phenomena encompass fog, rain, snow, turbulence, and solar irradiance. Notably, fog has been extensively studied due to its direct impact on V2V connections, as demonstrated in experimental simulations when compared to snow and rain [23,103,104,105].

On the other hand, other analytical studies showed that under conditions of dry ice, which has proven to have a more significant effect than fog on vehicle connection (with dense fog representing the most challenging environment), it was possible to obtain reliable communication according to the experimental measurements, reaching about 20 m. This was accomplished using image sensors at the receiver side, while the connection using PDs was about 15 m, which was the maximum distance achieved using other modulation schemes [103,104,105]. The effects of weather and smoke particles on visible short-range communications were examined and evaluated in [106]. Other factors with a negatively impact are fires, pollution, and smoke particles, which are also considered significant issues.

Smoke is one of the main challenges that must be considered, as the attenuation using the smoke model generated in the field of fire engineering is independent of wavelengths, as the highest field of view along the road is about five meters. “Visibility” refers to the maximum distance at which an object can be recognized apart from its background. In the context of two different external VLC settings, an investigation of the effects of fog and smoke was carried out. Moreover, the idea of visibility as a starting point for smoke and fog models uses the same kinds of equations to explain the optical attenuation, they induce to adjust the smoke and fog effect ratio. The channel attenuation coefficients were calculated using an energy simulator on the receiving side, considering the actual radiation pattern used in the LED, which reached about one kilometer [106,107].

## 5. Vehicular VLC Applications

Despite VLC vehicles having a limited communication range for transmitting waves, this technology has had comprehensive and advantageous implementation, especially in applications that require long distances to communicate with peers, such as in a platoon setting or within emergency implementations that need light electronic brakes from nearby peers, in addition to some intersection assistance implementations within a small area. On the other hand, vehicles can communicate out of range via VLC over the entire network or along a route; however, such cases require a longer latency so that messages are redirected through a multi-hop connection. As a result, vehicular VLC is not feasible if the application being considered must meet real-time requirements. Heterogeneous technologies with greater communication ranges (such as radio frequency (RF)) can be used to maintain contact for some implementations, especially outside the local area. The scaling needs for an application are implicitly impacted by the scope requirement (the number of vehicles required to connect). Therefore, VLC for vehicles can be used when there are an unlimited number of cars or there is the need to communicate with between vehicles within the line of sight. Regarding the necessity for the group structure, VLC vehicles can deal with other applications using either a short-range discontinuous or the long-range continuous method to complete the communication between cars. In theory, the virtual local coordination of vehicles can provide areas within the needs of limited real-time behavior. Therefore, a high delivery reliability is necessary to meet delivery time limitations.

However, in actual practice, it will become possible to connect with neighbors directly, especially if the optical channel has cases of interest to the user. Therefore, vehicular VLC should be explored as a supplementary communication channel for applications with strict reliability requirements to improve the system’s reliability and resilience. On the other hand, vehicular VLC is the only means of connection for implementations that can withstand network-related delays and accept best-effort connectivity applications for vehicular networks, which can be provided through vehicular VLC links alone or in a system of other heterogeneous networks. It is now possible to select these applications to be realized for employment within the VLC vehicle connection. The following are essential applications of vehicular VLC.

### 5.1. Lane Changing

A fundamental assumption in the majority of research on vehicular VLC systems is that two cars traveling in the same direction will perfectly align with each other. However, in practical scenarios, achieving perfect alignment is typically not feasible. Moreover, it is commonly believed that the leading car in a vehicular VLC system is equipped with one or two photodetectors mounted on its trunk. Even when two cars are traveling in the same lane, there will inevitably be some misalignment between them, regardless of whether they are positioned side by side. Similarly, maintaining a stable vehicular VLC connection during lane changes is crucial. One of the key design challenges in vehicular VLC systems is determining the minimum number of photodetectors (PDs) required and their optimal positions to ensure reliable communication in VLC-based V2V links, enabling continuous connectivity. Recent research has focused on addressing this challenge, as summarized in Table 4. These studies propose vehicular VLC methods that utilize PDs at the receiver side to enhance the VLC system while considering lane changes. For instance, in V2V VLC systems, it is common to assume the use of one or two PDs placed on the rear of the vehicles [36,39]. This configuration is typically sufficient for communication between two cars traveling in a straight line without horizontal displacement. However, for curved roads or wider roads (e.g., two-lane roads), additional PDs may be required to maintain effective communication.

For instance, in the study of [108], three PDs are employed, and the PD device with the highest received power is selected. In [109], a proposal is made to utilize four PDs in an angle diversity receiver, where the PDs are positioned in different directions. The work described in [37] focuses on maintaining V2V connectivity during lane changes on a straight two-lane road, employing a configuration with four PDs. On the other hand, ref. [38] takes it a step further by employing nine PDs for V2V and I2V VLC systems in order to achieve omnidirectional coverage at intersections and various road types. The proposed method was validated in different scenarios, including cruising in different lanes or the same lane, lane changes, and more. Regarding I2V systems, some studies, such as [35,80,110,111], concentrate on using a single PD, while others, like [80,110], investigate the location of the PD device on the front hood of the car. Certain works, such as [35,111], propose placing the PD at the top of the vehicle, citing its convenience for connecting with streetlights. The use of a single PD can be attributed to the presence of a clear line of sight on a single-lane road.

**Table 4:** A summary of works addressing vehicular VLC lane-change scenarios.

Refs.	Transmitter	Receiver	Scenario	Observations
[36]	Headlights	One PD	V2V	- Single lane - Straight road - Perfect alignment
[73,112]	Taillight	One PD	V2V	- Single lane - Straight road - Perfect alignment
[76]	Headlights	One PD	V2V	- Single lane - Straight road - Lateral shift
[32]	Headlight	One PD	V2V	- Two lanes - Straight road - Turning path
[113]	Headlights	One PD	V2V	- Three lanes - Straight road - Perfect alignment
[114]	Headlights	One PD	V2V	- Three lanes - Straight road - Lateral shift
[39]	Headlights	Twelve PDs	V2V	- Single lane - Straight road - Perfect alignment
[55]	Headlight and Taillight	Two PDs	V2V	- Three lanes - Straight road - Lateral shift
[108]	White LED	Three PDs	V2V	- Two lanes - Straight road - Turning path
[109]	Headlights	Four PDs	V2V	- Single lane - Straight road - One direction
[37]	Headlights	Four PDs	V2V	- Two lanes - Straight road - Omnidirectional coverage
[110]	Traffic Light	One PD	Hybrid I2V, V2I	- Single lane - Straight road - Perfect alignment
[80]	Traffic Light	One PD	I2V	- Single lane - Straight road - Perfect alignment
[111]	Streetlight	One PD	I2V	- Two lanes - Straight road - Perfect alignment - Channel estimation
[35]	Streetlight	One PD	I2V	- Two lanes - Straight road - Lateral shift
[38]	Headlights and Taillights Streetlight and Traffic Light	Nine PDs	V2V and V2I	- Omnidirectional coverage - Multi-lane and curved roads - Intersections - Perfect alignment - Misalignment - Lateral shift

### 5.2. Intersection Assistance

Assistance at intersections is one of the main applications of vehicular VLC. It helps avoid collisions at intersections, making them safer. Alternative means of warning and coordination between vehicular communications are provided, in addition to the traditional method represented by a traffic light. The collision avoidance system at intersections is one of the primary examples of the assistance applications at a corner, where the vehicles face each other. Thus, it is possible to take advantage of the VLC links for the cars for face-to-face communication and also with the vehicles parked on the other side of the intersections. In addition, the traffic lights provide communication based on LEDs or through other components, which makes connection easier.

**Figure 7:**
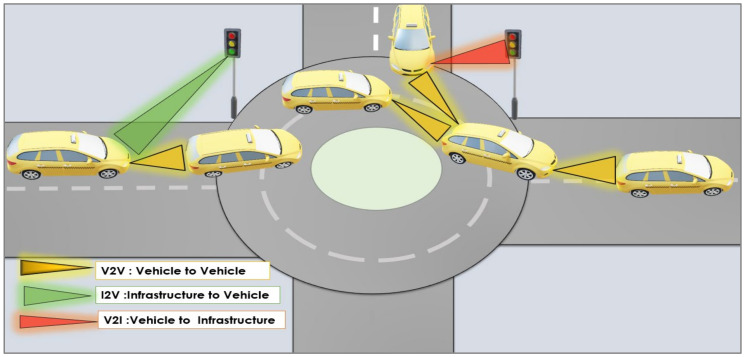
Intersection assistance structure for V2I, I2V, and V2V.

Recently, the development of intersections has seen increasingly complex forms. Despite their complexity, paths can be redesigned by applying guidance and navigation methods and combining knowledge to control road traffic. Figure 7 shows the communication between vehicles (V2V) and between infrastructure and vehicles (I2V) or from vehicles to infrastructure (V2I) via streetlamps and traffic lights to exchange information. Vehicles use headlights and taillights in addition to traffic lights, which allows data privacy and digital safety to be preserved. Many works [23,38,115,116,117] have discussed the topic of intersection assistance applications.

For example, in [115], it was suggested that the data be encoded in transmitters and converted into light signals. A silicon/carbon wavelength division multiplexer is used in the receivers for decoding. I2V, V2I, and V2V traffic scenarios are generated and then developed to control the arrival of vehicles at the intersection and schedule them to cross at times that minimize delays and maintain a safe distance from one to the other. This work in [116] proposed using hybrid VLC and RF to deploy important safety messages (BSMs) in intersection applications instead of conventional vehicle RF communication. Moreover, relaying BSMs is also compared, where outage, latency, throughput, and performance improve dramatically. In [117], the authors proposed the use of microsimulation and traffic-controlled intersection analysis, using a mesh-cellular hybrid network configuration for a vehicle-to-everything (V2X) scenario, the request/response concept, and relative position estimation for route management, as well as a wave-length-division multiplexer (WDM) optical sensor in the receiver. The redesigned intersection layout and collaborative I2V and V2V messaging are important issues in controlling traffic flow.

### 5.3. Platooning

The widespread availability of LEDs in vehicles has attracted the interest of many researchers, as these LEDs are used in current studies of headlights, brake lights, and turn signals for Infrastructure-to-Vehicle (I2V) and Vehicle-to-Vehicle (V2V) communications [23,68]. The faction support app is among the many attractive applications for VLC-based ITSs. The platoon implementation supports mutual data type requirements and a relatively short connection link [118]. Moreover, the platooning approach provides increased throughput on highways and urban areas and improves traffic safety. In the most common methods using the platoon approach, the existing conventional radio frequency technology is often the solution for exchanging messages between platoon members. Although the traditional frequency of radio communication technologies provides relatively long-range communication and high data rates and shares the same spectrum for vehicular applications, these technologies face some challenges and limitations in high-density traffic scenarios due to RF channel congestion [23].

**Figure 8:**
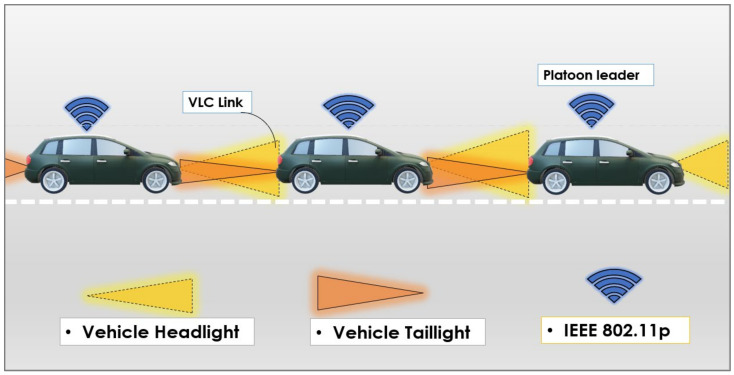
Platoon V2V VLC architecture.

VLC has recently been utilized in ITSs for platoon applications. It can complement other networks such as C-V2X and IEEE 802.11p to facilitate communication and information exchange between the platoon leader and the other vehicles within the platoon, as depicted in Figure 8 [119]. An analytical modeling of VLC is presented in [118] to define the key parameters of a platoon application and demonstrate its performance. The outdoor VLC system also incorporates a lateral and longitudinal platoon controller development approach. The lateral control unit aims to track the vertical vehicle path between V2V VLC connections, while the longitudinal control unit maintains a constant distance between vehicles. Maintaining LoS continuity is one of the most challenging scenarios in vehicular applications, particularly for ITS. In order to address this challenge, information exchange is crucial among platoon members regarding relative directional location, including the forward and rear-facing directions of each vehicle. In [120], a compensation approach using four cars equipped with VLC and positioning systems is proposed. Different scenarios are simulated to examine the limits of compensation angles and varying trajectories. Furthermore, ref. [121] presents testing of a VLC system in several configurations using headlights and commercial taillights, focusing on their compatibility within a platoon. The system achieves a bit error rate (BER) of less than 10^−6^ at a distance of 30 m and a data rate of approximately 100 kbps, with a latency of 4.2. The system’s performance is also assessed in the presence of a jamming vehicle, demonstrating that it does not cause deterioration while crossing the platoon.

## 6. Vehicular VLC Channel Modeling and Propagation Characteristics

### 6.1. Key Parameters of Vehicular VLC

A. Asymmetrical pattern

In general, the main feature of the vehicular VLC is the variable intensity of the headlights and taillights (i.e., asymmetry) [114,122,123]. In addition, the headlights and taillights in the vehicular VLC serve two purposes simultaneously: illuminating and transmitting information. Hence, the intensity of the light emitted from the headlights is several times stronger than that of the taillights. In contrast, it creates an asymmetrical association between vehicles with this light unit. Moreover, the intensity of illumination of the headlights of cars is irregular. On the other hand, the ideal Lambert model is widely used for modeling VLC channels and obtaining CIRs. In this method, any light source is assumed to have a uniform radiation pattern, with the intensity of the light defined as a function of the radiation angle (α). $n_{L}=-\ln(2)/\ln(cos\theta_{\frac{1}{2}})$ must be in the Lambertian sequence at the half-power angle $\theta_{1/2}\;$. The channel impulse response (CIR) can be calculated using (*β*) angle of incidence and propagation distance (*d*) [124]:

(2) $$h\left(t\right)=\frac{{(n}_{L}+1)\;A}{2\pi d^{2}}\;{cos}^{n_{L}}(\alpha )\;cos(\beta )\delta (t-\frac{d}{c}\;)$$

where (*A*) denotes the area of the photodetector and (*c*) represents the speed of light. It should be noted, however, that this ideal model should be only used for indoor VLC scenarios due to several reasons. For instance, this ideal model does not consider that the vehicular light sources have a symmetrical radiation pattern [29] that is designed to achieve specific illumination functions following the road regulations.

Additionally, outdoor lights, such as HLs [76] and TLs [112], have asymmetrical radiation patterns because they are intended for different illumination purposes. The Lambertian model fits well with most indoor light sources. Still, it does not work well with outdoor light sources. Moreover, experimental measurements or non-sequential ray tracing methods can be used where the measured radiation pattern considers the properties of outdoor light sources. The channel impulse response (CIR) is calculated using the following equation [125]:

(3) $$h\left(t\right)=\sum_{K=1}^{M}P_{K}\delta (t-\tau_{K}),$$

where *M* denotes the total ray number captured within the photodiode, δ denotes the Dirac delta function, $P_{K}$, and $\tau_{K}$ respectively represent power and delay in the transmission of the signal of the $k^{th}$ ray, and *k* = 1, 2, *M*.

The PL is given by

(4) $$PL=10{log}_{10}[\int_{0}^{\infty }h\left(t\right)dt]$$

B. Mobility

Another critical parameter for VLC technology is mobility, due to the required Line of Sight (LoS). A precise spatial alignment is required between the optical devices transmitting and receiving data in a VLC link. This is because classic optical receivers that use photodiodes offer a field of view (FOV) or narrow cones on the receiver side. Performing an alignment of this nature becomes a challenging task. It is possible to enlarge the field of view on the side of the receiver by placing a lens in front of the receiver components, but to do so will compromise the work of the VLC system due to the increase in the amount of received noise, as the system must collect this noise, and preserve the field of view, and reduce the proportion of this noise spatially by using reception components inspired by the camera’s components [34,38].

On the other hand, selective filtering in the receiver improves the signal-to-noise ratio of the background noise in the environment. The spatial solution allows the dotted structure to filter this ambient noise. Thus, the large field of view gives both the receiver and the transmitter great flexibility in their mobility. In this manner, camera-inspired receivers present a variety of distinct benefits to address the problem of mobility. Moreover, to solve the mobility issue, one must first thoroughly comprehend the many motions the vehicular VLC system could experience, both in magnitude and type. The movement of vehicles is characterized by a model representing the relative placement along all three spatial dimensions of two cars (the transmitter and the receiver). The following identifies the motion model as a vector [34,37,38]:

(5) M=[δu δv δw n]

where *δu* represents the relative movement along the horizontal axis (*X*), δv represents the relative movement along the vertical axis (*Y*), δw denotes the relative motion within the length of the driving path (*Z*), and n indicates the value of the flag, which represents the type of vehicle behavior class. This section describes the motion model for specific time slots.

The values of relative movement illustrate the movement that occurred during a specific period. The vehicle action category describes the type of movement experienced by the vehicle during that particular time window. In addition, the influences and ability of the V2V VLC approach must be considered within the several output applications, which leads to a significant deterioration in the completion of the communication link. The transformation needs to consider the fact that there is a substantial increase in environmental noise. There must be a lateral separation of the vehicle and a longitudinal separation between the two connected cars, which must be considered since there are parked or moving vehicles near the two connected cars. Moreover, since the LED for the VLC is mainly used with low-range communications, the longitudinal separation method significantly impacts the connection of the two vehicles to each other, affecting the system’s ability to work effectively [37,38,124].

C. Weather conditions

Currently, there is a lack of research exploring the impact of changing weather conditions, such as rain, snow, and fog, on vehicular VLC systems. Limited studies exist on this topic [39,76,103,104,105]. Weather variations pose significant challenges for vehicular VLC, and it is challenging to conduct empirical trials to accurately characterize the performance of vehicular VLC under different weather conditions. In the existing literature focusing on weather studies and modeling of the communication channel in vehicular VLC, two well-established models are utilized. One is the Mi theory model [126], which is commonly employed for studying wireless communication in photovoltaic cells, particularly when lasers are involved. The other is the Kim theory model [127]. These models serve as frameworks for understanding the effects of weather on vehicular VLC and are adapted from related fields of wireless communication and photovoltaic cell research.

These paradigms make it possible to calculate the coefficient factor of the atmospheric attenuation for foggy conditions. This coefficient has been generalized for rain and snow, since it is connected to the atmospheric visibility parameter [75,104]. The most challenging aspect of utilizing this attenuation model is locating the appropriate physical parameters (such as the amount of liquid water present, the distribution of particle sizes, the average particle size, and the reduction in visibility), which accurately describe certain weather phenomena [128].

Furthermore, when simulating the meteorological conditions, it is essential to consider the indirect effects of these factors on communication. Even though poor weather conditions almost always make it more difficult to communicate via LoS, there are situations in which they can increase the reflectivity of the ground (such as when it rains or snows, and the roads become wet), which in turn makes it easier to communicate via NLoS. This problem has been taken into consideration by some researchers [36,39,76], who have used a CAD tool to describe reflected aspects of the surroundings (for example, the road surface and automobiles). In addition to the physical properties necessary to photograph rain and fog influences (based on the theory of Mi scattering), atmospheric cases affect the kinds of sediment that accumulate on light equipment. This affects the amount of light emitted by that light unit. The attenuation of different wavelengths by the atmosphere under a range of other atmospheric conditions is given in Table 5 [129].

D. Receiver aperture size

Vehicular VLC systems employ two primary methods for receiving light signals transmitted by vehicle headlights and taillights: camera image sensors and PDs. These devices capture the received light signal and convert it into an electric current signal, facilitating subsequent decoding and demodulation processes. The utilization of both camera-based and PD-based receivers has been extensively documented in vehicular VLC research [17,29,130,131,132]. Camera-based and PD-based receivers are considered due to their distinct design and engineering characteristics, as well as the differences in how they capture and process optical signals. These differences significantly influence the overall architecture and performance of vehicular VLC systems. Various aspects, such as media access control, modulation techniques, coding schemes, noise reduction, and interference mitigation, are tailored based on whether the receiver utilizes an image sensor or a PD.

Typically, two types of PDs are used in vehicular VLC: Avalanche Photodiode (APD) and PIN (p-n diode). The APD devices have better gain and higher sensitivity than PIN PDs. Moreover, a type of APD with higher sensitivity, such as a Single-Photon Avalanche Diode (SPAD), works better in simulation than traditional APD devices, especially in bad weather conditions, at a lower cost, in addition to having temperature tolerance. The main point of the vehicular VLC systems when choosing a PD receiver is that the characteristics of the receivers should be taken into account to achieve optimal performance, such as suitable bandwidth, low noise, high sensitivity, and wide linear range; the main factor is the size of the active area of the PD device. All these parameters significantly affect the performance of the vehicular VLC systems. For example, when the size of the PD receiving area is large, or the field of view (FOV) is large, it will allow the capture of a large number of optical signals in addition to allowing the reception of other unwanted signals, which causes system degradation and thus reduces the signal-to-noise ratio (SNR) [17,18,133]. Researchers and academics have considered the effect of the active area size parameter for vehicular VLC [37,38,56,69,84,134,135]. It is possible to improve VLC receiver performance by utilizing an optical filter. It is also possible to reduce the impact of interference by limiting the FOV on the side of the recipient device. However, it will significantly influence the service area [11]. The path loss, considering the active aperture area of PD, can be calculated as follows:

(6) $$PL=10\;log\left(A_{r}\left(m+1\right)\right)-10\;log(2\pi )-20\;log\;D+10(m+1)log\;cos(\varphi )$$

where *φ* denotes the irradiance azimuth (horizontal) angle, *ψ* denotes the angle of an incident at the recipient side, *D* denotes the distance between vehicles, *Ar* represents the active aperture area$,{\;\;\Psi }_{1/2}$ indicates the {half-power angle} of radiation, and $\{m=\frac{-0.6931}{\ln\left(\cos\left(\Psi_{\frac{1}{2}}\right)\right)}\}\;$denotes the index of Lambertian order.

### 6.2. Existing Vehicular VLC Channel Models

This section discusses the existing vehicular VLC channel models for V2V with headlights and taillights and I2V with either streetlights or traffic lights.

1. V2V with High-Beam Headlights

- Linear model

For perfect alignment [39], the linear model of path loss between vehicles VLC can be written as

(7) $$P_{r\;}=P_{t}(\alpha d+\beta )$$

where $P_{r\;}$represents the received optical power, $P_{t}$ represents the transmission power, *α* and β represent weather coefficients, and d represents the inter-vehicle distance.

- Exponential Model

The exponential path loss for vehicle-to-vehicle VLC can be written as

(8) $$Pr=Pt\;\underset{p}{\underbrace{Ad^{-2B\;}\exp(-cd)}}$$

where Pt represents the transmission power, A represents the geometrical loss value at a reference distance specified as $d_{0}$, B is the decaying factor as determined by {$A=P_{0\;}{d_{0}}^{2B}\exp\left(cd_{0}\right)/Pt\}$, c represents the extinction coefficient for clear, rainy, and foggy weather, and p is the optical channel coefficient.

- Comprehensive Model

For lateral shift and receiver aperture effect, we can obtain the overall path loss as [76] $PL=10{log}_{10}$

(9) $$\frac{1}{2}\sum_{i=1}^{2}\left({\left(\frac{D_{R}{\left(d/\sqrt{d^{2}+{d_{h_{i}}}^{2\;}}\right)}^{\frac{1}{\varepsilon }}}{\varsigma \sqrt{d^{2}+{d_{h_{i}}}^{2\;}}}\right)}^{2}exp\left(-c\sqrt{d^{2}+{d_{h_{i}}}^{2\;}}\;{\left(\frac{D_{R}}{\varsigma \sqrt{d^{2}+{d_{h_{i}}}^{2\;}}}\right)}^{\varepsilon /2}\right)\right)\;$$

where $D_{R}$ represents an aperture diameter, ζ and ε are two correction coefficients that consider the pattern of the headlamp light to be asymmetrical, in addition to the weather conditions, $\sqrt{d^{2}+{d_{h_{i}}}^{2\;}}$ represents the distance of propagation between the $i_{th}$ TX, *i* = 1, 2, and RX.

2. V2V with Low-Beam Headlight

The optical channel model for the asymmetric low beam headlight pattern ($h_{opt}$) is given as

(10) $$h_{opt}[dB]\approx 10{log}_{10}{\left(\frac{4D_{R}}{3\left(d+d_{h}^{2}\right)}\right)}^{2}$$

where $D_{R}$ refers to the diameter of the power meter’s effective area, $d_{h}$ represents stands for the lateral displacement, and d represents stands for the distance between vehicles.

Based on computation, it was determined that the loss of the geometric shape dominates the failure of the trajectory. Additionally, because the beam is parallel, the lateral distance has a more significant influence than the distance between vehicles by a factor of two [31]. It is essential to emphasize the fact that the model relies on there being a low-beam headlight present in the vehicle. The photodetector on the receiving vehicle is perfectly aligned with the headlight that is being assessed, and there is no evidence of any lateral displacement.

3. V2V with Taillights

The formula for the path loss model, an explicit function of both d and the distance from vehicle to vehicle in terms of its taillights, is as follows [73]:

(11) $${PL}_{i}\left(d\right)={PL}_{0,i}+10{log}_{10}\left(d^{-2\varepsilon_{i}}\right)-\zeta_{i}d,i\in \left\{a,b,c\right\}$$

where ${PL}_{0}$ represents the reference PL at $d_{0}$ of one meter, and ${\zeta \;}_{i}$ and $\varepsilon_{i}$ represent correction coefficients values.

4. I2V with Traffic Lights

The model of loss of the infrastructure to vehicle (I2V) connection with traffic lights is given as follows [135]:

(12) $${PL}_{I2V}=10{log}_{10}\left(h_{I2V}\right)=a\;exp\left(-{\left(\frac{d_{I2V}-b}{c}\right)}^{2}\right)$$

where $h_{I2V}$ denotes the optical channel coefficient, equal to the loss computed on a linear standard; {*a*, *b*, and *c*} are denoted coefficients that were acquired using ray tracing for each $d_{h}$ (which demonstrate the horizontal shift that occurred within I2V).

5. I2V with Street lights

Due to the regular distribution of streetlights over a distance and the assigned area between them, a car traveling approaches a lamp and then passes away from it. As a result, the received energy shows a periodic pattern (sinusoidal behavior), and the closed form of the path loss equation of the model can be defined as follows [83]:

(13) $$h_{dB\;}=C_{P}z+C_{SA}$$

where$\;z=cos((2/d_{T})d)$, *d* is the distance the vehicle travels from one streetlamp to another, 2$C_{P}$ and $C_{SA}$ are denoted as the peak-to-peak change in received power and the sinusoidal axis by the notation, respectively.

$C_{P}$ and $C_{SA}$ can be determined for any given scenario by
  (14) $$C_{P}=0.5f\left(d_{y\;},D_{r},H_{D},S_{p},b_{1}\right)-0.5\left(d_{y\;},D_{r},H_{D},S_{p},b_{2}\right),$$
  (15) $$C_{SA}=0.5f\left(d_{y\;},D_{r},H_{D},S_{p},b_{1}\right)-0.5\left(d_{y\;},D_{r},H_{D},S_{p},b_{2}\right),$$
  Here,
  $f\left(d_{y},D_{r},H_{D},S_{p},b_{i}\right)$, *i* = 1,2 is defined as
  (16) $$\begin{array}{cc} f\left(d_{y\;},D_{r},H_{D},S_{p},b_{i}\right) & \\ & =D_{r}\left(b_{i,1}+b_{i,2}d_{y\;}+b_{i,3}H_{D}+b_{i,4}S_{p}+b_{i,5}D_{r}\right)\\ & +d_{T}\left(b_{i,6}+b_{i,7}d_{y\;}+b_{i,8}H_{D}+b_{i,9}S_{p}\right)\\ & +H_{D}\left(b_{i,10}+b_{i,11}d_{y\;}+b_{i,12}H_{D}\right)+d_{h}\left(b_{i,13}+b_{i,4}d_{y\;}\;\right)+b_{i,15} \end{array}$$
  where dy is the lateral shift function, *Dr* is the receiver aperture, $H_{D}$ is the height difference between streetlamp and receiver, $S_{p}$ is the separation between streetlight poles, and $b_{i,j}$ is the $j^{\mathrm{th}}$ element of $b_{i}$.

## 7. Vehicular VLC Research Trends and Future Directions

In this section, we discuss various relevant research topics for vehicular VLC systems, highlight some lessons learned, and illustrate some future research directions.

A. Research Trends

- Multi-hop Relaying

The performance of vehicular VLC systems is heavily dependent on LoS links, which may not always be feasible in practical scenarios. Consequently, several crucial technologies have emerged to address this challenge. One key enabler for connectivity in vehicular VLC is multi-hop transmission, where the signal transmitted from a source car can reach a destination car through a series of intermediate cars known as relays. The authors of [36] examined the deployment of relay-assisted V2V systems using a linear path loss model to extend transmission ranges. Moreover, the authors in [136] analyzed the error rate and coverage range of multi-hop V2V VLC systems under different weather conditions, employing a more realistic channel model. Their findings confirmed that multi-hop relaying can significantly enhance performance. In [118], focused on a VLC-based platoon, incorporating longitudinal and lateral control to maintain a constant inter-distance between cars. Cailean et al. presented experimental results for a dual-hop vehicular VLC system in [135]. Their work aimed to connect a distant car to a roadside access point through a closer car. Eldeeb et al. explored the medium access control (MAC) layer performance of multi-hop vehicular VLC networks using carrier sense multiple access (CSMA) protocols in [55]. To enhance connectivity in an I2V link, the authors in [137] proposed a relay-assisted approach, where streetlamps can act as access points and other vehicles can function as nodes. This setup helps maintain communication links in NLoS conditions.

- Reconfigurable Intelligent Surfaces

Another promising technology is reconfigurable intelligent surfaces (RISs), which show great potential in mitigating LoS constraints in vehicular VLC systems. While there is an increasing body of research on RISs in indoor VLC, investigations into the performance limits and advantages of RISs in outdoor VLC are still in their early stages. For instance, in [138], researchers explored the signal-to-noise ratio (SNR) with varying numbers of mirrors and transmission distances. They also employed an exhaustive search method to determine the maximum distance between adjacent RISs. In [139], the authors examined the outage probability, throughput, and delay outage rate of an RIS-aided hybrid VLC/RF V2V system, although the RIS was only considered for the RF link. In [140], an optimization algorithm was utilized to determine the optimal number of mirrors that maximize energy efficiency while satisfying data rate and bit error rate requirements. Recently, in [141], researchers derived closed-form expressions for energy efficiency and spectral efficiency in RIS-assisted V2V VLC systems. The effect of random misalignments was then considered in [142]. These findings demonstrated the significant performance enhancements made possible by RIS technology in vehicular VLC systems.

B. Lessons Learned

The concept of vehicular VLC is a crucial enabler for ITSs to improve road safety, traffic flow, and fuel consumption efficiency. Realistic channel models are considered the first step in investigating the performance of vehicular VLC systems.

- Earlier results have focused on indoor channel modeling, which does not apply to vehicular VLC systems with inherently different characteristics. For example, earlier works assumed the ideal Lambertian model for vehicular light sources, which fails to match the illumination characteristics of automotive headlights, taillights, traffic lights, and streetlights, with their asymmetrical intensity distributions. Any modification of the antenna pattern will significantly affect the communication performance.
- The effects of road reflectance, road type, weather conditions, the orientation of the user/vehicle equipment and infrastructure, receiver aperture size, and the sunlight might strongly affect the link performance of vehicular VLC systems. Also, vehicular mobility can result in LoS blockage, and thus, assisting technologies such as relaying techniques and RISs should be utilized. It is paramount to investigate the effects of these factors on traffic safety based on realistic channel models.

C. Future Directions

In the future, several technologies and applications based on vehicular VLC should be explored to achieve safe conditions for ITSs. For instance, digital twin technology has been recently introduced to enable smart, connected, and automated vehicles [142,143,144,145,146,147,148,149,150]. The authors of [148,150] discussed its applicability in vehicular VLC systems and its ability to provide effective improvement, meeting various requirements for emerging applications. Digital twin-assisted vehicular VLC systems can also help in optimizing the phase shifts of RIS elements installed in the infrastructure of roads, cars, bus stations, and buildings [150]. Another promising research direction is the applications of artificial intelligence and machine learning algorithms in vehicular VLC, which can help in the optimization and security of vehicular VLC systems. Integration between vehicular VLC networks and mm Wave networks is also considered an open research direction to further ensure continuous connectivity and low latency.

## 8. Conclusions

Vehicular VLC plays a crucial role in ITSs by facilitating information exchange between vehicles and infrastructure to achieve key objectives, such as improving road safety, passenger comfort, and traffic flow. This study encompassed a comprehensive literature review covering the general architecture of VLC and its applications in vehicular communication, including platooning, intersection assistance, and lane-change applications. The study also highlighted the main challenges faced by vehicular VLC systems, such as the influence of road reflections, noise from sunlight and artificial lighting, and varying weather conditions. Furthermore, critical parameters for vehicular VLC channel modeling were discussed, including the asymmetrical pattern of vehicular light sources, vehicle mobility, and the atmospheric propagation medium. Moreover, the existing vehicular VLC channel models for V2V communication utilizing headlights and taillights, as well as I2V communication involving streetlights or traffic lights, were examined. Furthermore, some key directions were highlighted, including the utilization of digital twin technology and the applications of AI and ML algorithms in vehicular VLC systems, showcasing their potential to optimize resource allocation, enhance security, and enable intelligent decision making. Finally, we provided insights into the integration of vehicular VLC with mm Wave networks as an open research topic, highlighting its potential for achieving continuous connectivity, low latency, and advanced vehicular services. This survey aims to serve as a valuable reference and guide for both experts and newcomers in the field of vehicular VLC.

## Figures and Tables

**Table 1:** Surveys on visible light communication.

Content Explored		[18]	[19]	[20]	[21]	[22]	[23]	[24]	[25]	[26]	[27]	This Survey
Superiority of VLC		√	√	√	√	√	√	√	√	√	√	√
Architecture	Transmitter	√	√	√	√	√	√	√	√	√	√	√
	Receiver	√	√	√	√	√	√	√	√	√	√	√
	Channel modeling			√		√	√		√	√	√	√
Modulation	OOK, PWM, PPM	√		√		√	√	√	√		√	√
	OFDM			√		√		√	√		√	√
Challenges	LoS	√		√		√	√		√	√		√
	Noise and Interference	√	√		√	√	√		√	√		√
	Mobility	√		√	√		√		√			√
VLC Application	Indoor	√	√	√	√	√		√	√	√	√	√
	Positioning	√		√	√			√			√	√
	Smart cities		√									√
	Hospital		√	√					√			√
	Vehicular	√	√	√			√	√	√	√	√	√
	Underwater	√						√		√	√	√
Processing Time		√		√	√		√		√	√		√
Computational Complexity				√	√	√			√	√		√
Energy efficiency		√	√	√	√	√	√	√	√	√	√	√

**Table 2:** Vehicular VLC light sources and channel modeling summary.

Ref.	Year	Application	Light Source	Channel Method	Observations
[72]	2018	V2V	Lambertian	GBSM	The authors considered the following: - Line-of-sight (LoS) received power; - Effective scatterers on the Tx and Rx; - Single and double-bounced rays; - Lambertian light source, channel gain; - Different distance ranges; - PD is located at three different heights.
[36]	2018	V2V	Asymmetrical	Ray tracing	- Weather conditions (fog, rain); - Road reflection, achievable distance; - Asymmetrical headlight; - Multi-hop transmission; - Simulation built-in ray tracing.
[75]	2019	V2V	Lambertian	Experimental	- Fog conditions; - LED in TX and camera in RX; - Experimental setup; - Effect of visibility levels; - The modulation index (MI).
[39]	2019	V2V	Asymmetrical	Ray tracing	- Weather conditions (clear, rainy, foggy); - Dual photodetector; - Asymmetrical high-beam headlamp; - Achievable transmission distance; - Simulation built-in ray tracing.
[37]	2019	V2V	Asymmetrical	Ray tracing	- Various scenarios: (imperfect alignment, lane changes); - Four photodetectors; - Asymmetrical high-beam lights; - Achievable power percentage of each PD; - Simulation built-in ray tracing.
[74]	2020	V2I	Asymmetrical	Ray tracing	- Impact of optical CIRs; - The effect of lateral shifts; - Three photodetectors (traffic pole); - Two-lane road; - Two asymmetrical headlamps (TX); - Achievable data rate; - Achievable SNR; - Simulation built-in ray tracing.
[76]	2020	V2V	Asymmetrical	Ray tracing	- Obtain the channel impulse (CIRs); - Two high-beam headlamps; - Investigate the maximum transmission; - Various weather conditions (clear, rainy, foggy); - Transmitter beam divergence angle; - Receiver aperture diameter; - Lateral shift; - Simulation built in non-sequential ray tracing.
[35]	2020	I2V	Asymmetrical	Ray tracing	- Two-lane road; - A single PD located on the top of a car; - A streetlamp with asymmetrical light; - Effect of blockage on handover; - CIR calculation and analysis; - Simulation in non-sequential ray tracing.
[80]	2020	I2V	Lambertian	Experimental	- Analyzing Field of View (EFOV); - Calculate Packet Error Rate (PER); - Analyzing an optical channel model; - An empirical analysis test; - Lambertian model of traffic light LED; - Obtain minimize latency; - Effects of non-LoS.
[73]	2021	V2V	Asymmetrical	Ray tracing	- Obtain the channel responses (CIRs); - Derived path loss mode; - Obtain BER; - Investigate RMS delay spread; - Use different commercial taillights.
[81]	2021	I2V	Lambertian	Experimental	- Channel characterization for I2V; - Real traffic light in a typical urban road; - The Lambertian channel model; - Receiver is located at 3 different heights; - Experimental measurement setup; - Obtain bit error rate (BER); - Measure signal-noise-ratio (SNR).
[83]	2022	I2V	Asymmetrical	Ray tracing	- Obtain BER performance; - Derive a closed-form expression; - Asymmetric LED light TX; - Obtain minimize latency; - Receiver aperture of PD; - Lateral shift and height between I2V; - Longitudinal distance between I2V.

**Table 3:** The effect of LoS, NLoS, and reflections on vehicular VLC.

Method	Refs.	Scenario	Comments
**LoS**	[69]	V2V	Proposed a headlight LoS path loss model
	[74]	V2V	Submitted a taillight LoS loss model
	[30]	V2V	Presented a LoS path loss model for an asymmetric headlight
**NLoS**	[98,99]	V2V	Described the effects of reflections on road surfaces
	[96]	V2V	Reported on specular reflections on the road surface
	[97]	V2V	Focused on the reflection from surrounding vehicles

**Table 5:** Attenuation of incoming electromagnetic waves by the atmosphere under a variety of weather conditions and wavelengths [129].

Weather Condition	Visibility (km)	λ = 785 nm Attenuation (dB/km)	λ = 850 nm Attenuation (dB/km)	λ = 1550 nm Attenuation (dB/km)
Clear air	23	0.5	0.4	0.1
Haze	2	6.7	6.4	4.2
Light fog	0.8	19.1	18.6	15.5
Moderate fog	0.6	27.3	27	25.5

## Abbreviations

BER	Bit Error Rate
CAD	Computer-Aided Design
CIR	Channel Impulse Response
DSRC	Dedicated Short-Range Communication
DD	Direct Detection
FOV	Field of View
HLs	Headlights
I2V	Infrastructure-to-Vehicle
ITS	Intelligent Transportation System
LED	Light-Emitting Diode
LoS	Line-of-Sight
LTE	Long-Term Evolution
NLoS	Non-Line-of-Sight
OWC	Optical Wireless Communication
RMS	Root mean square
OOK	On–off keying
PWM	Pulse width modulation
PPM	Pulse position modulation
OFDM	Orthogonal frequency division multiplexing
PD	Photodetector
PDF	Probability Distribution Function
RF	Radio Frequency
SNR	Signal-to-Noise Ratio
TLs	Taillights
V2X	Vehicle-to-Everything
V2V	Vehicle-to-Vehicle
VLC	Visible Light Communication
V-VLC	Vehicular Visible Light Communication

## References

[1] Pribyl O., Pribyl P., Lom M., Svitek M. (2018). Modeling of smart cities based on ITS architecture. IEEE Intell. Transp. Syst. Mag..

[2] Miucic R. (2019). Connected Vehicles. Intelligent Transportation Systems.

[3] Chen S., Hu J., Shi Y., Peng Y., Fang J., Zhao R., Zhao L. (2017). Vehicle-to-everything (V2X) services supported by LTE-based systems and 5G. IEEE Commun. Stand. Mag..

[4] MacHardy Z., Khan A., Obana K., Iwashina S. (2018). V2X access technologies: Regulation, research, and remaining challenges. IEEE Commun. Surv. Tutor..

[5] Ghafoor K.Z., Guizani M., Kong L., Maghdid H.S., Jasim K.F. (2019). Enabling efficient coexistence of DSRC and C-V2X in vehicular networks. IEEE Wirel. Commun..

[6] Cai X., Peng B., Yin X., Yuste A.P. (2017). Hough-transform-based cluster identification and modeling for V2V channels based on measurements. IEEE Trans. Veh. Technol..

[7] Gao S., Lim A., Bevly D. (2016). An empirical study of DSRC V2V performance in truck platooning scenarios. Digit. Commun. Netw..

[8] Shen W.H., Tsai H.M. Testing vehicle-to-vehicle visible light communications in real-world driving scenarios. Proceedings of the 2017 IEEE Vehicular Networking Conference (VNC).

[9] Vivek N., Srikanth S.V., Saurabh P., Vamsi T.P., Raju K. On field performance analysis of IEEE 802.11 p and WAVE protocol stack for V2V & V2I communication. Proceedings of the International Conference on Information Communication and Embedded Systems (ICICES2014).

[10] Xu Z., Li X., Zhao X., Zhang M.H., Wang Z. (2017). DSRC versus 4G-LTE for connected vehicle applications: A study on field experiments of vehicular communication performance. J. Adv. Transp..

[11] Gerhátné Udvary E. (2019). Visible light communication survey. Infocommunications J..

[12] Aghaei F., Eldeeb H.B., Bariah L., Muhaidat S., Uysal M. (2023). Comparative Characterization of Indoor VLC and MMW Communications via Ray Tracing Simulations. IEEE Access.

[13] Eldeeb H.B., Hosney M., Elsayed H.M., Badr R.I., Uysal M., Selmy H.A.I. (2020). Optimal Resource Allocation and Interference Management for Multi-User Uplink Light Communication Systems with Angular Diversity Technology. IEEE Access.

[14] Karunatilaka D., Zafar F., Kalavally V., Parthiban R. (2015). LED based indoor visible light communications: State of the art. IEEE Commun. Surv. Tutor..

[15] Cailean A.M., Dimian M. (2017). Impact of IEEE 802.15. 7 standard on visible light communications usage in automotive applications. IEEE Commun. Mag..

[16] (2019). Car Lighting District. Halogen vs. HID vs. LED—Which is Best?. https://www.carlightingdistrict.com/.

[17] Liu C.B., Sadeghi B., Knightly E.W. (2011). Enabling vehicular visible light communication (V2LC) networks. Vehicular Inter-Networking.

[18] Matheus LE M., Vieira A.B., Vieira L.F., Vieira M.A., Gnawali O. (2019). Visible light communication: Concepts, applications and challenges. IEEE Commun. Surv. Tutor..

[19] Rehman S.U., Ullah S., Chong PH J., Yongchareon S., Komosny D. (2019). Visible light communication: A system perspective—Overview and challenges. Sensors.

[20] Lian J., Vatansever Z., Noshad M., Brandt-Pearce M. (2019). Indoor visible light communications, networking, and applications. J. Phys. Photonics.

[21] Rahman A.M., Li T., Wang Y. (2020). Recent advances in indoor localization via visible lights: A survey. Sensors.

[22] Ibhaze A.E., Orukpe P.E., Edeko F.O. (2020). High capacity data rate system: Review of visible light communications technology. J. Electron. Sci. Technol..

[23] Memedi A., Dressler F. (2020). Vehicular visible light communications: A survey. IEEE Commun. Surv. Tutor..

[24] Shaaban K., Shamim MH M., Abdur-Rouf K. (2021). Visible light communication for intelligent transportation systems: A review of the latest technologies. J. Traffic Transp. Eng. (Engl. Ed.).

[25] Abuella H., Elamassie M., Uysal M., Xu Z., Serpedin E., Qaraqe K.A., Ekin S. (2021). Hybrid RF/VLC systems: A comprehensive survey on network topologies, performance analyses, applications, and future directions. IEEE Access.

[26] Yahia S., Meraihi Y., Ramdane-Cherif A., Gabis A.B., Acheli D., Guan H. (2021). A survey of channel modeling techniques for visible light communications. J. Netw. Comput. Appl..

[27] Geng Z., Khan F.N., Guan X., Dong Y. (2022). Advances in Visible Light Communication Technologies and Applications. Photonics.

[28] Cailean A., Cagneau B., Chassagne L., Topsu S., Alayli Y., Blosseville J.M. Visible light communications: Application to cooperation between vehicles and road infrastructures. Proceedings of the 2012 IEEE Intelligent Vehicles Symposium.

[29] Căilean A.M., Dimian M. (2017). Current challenges for visible light communications usage in vehicle applications: A survey. IEEE Commun. Surv. Tutor..

[30] Alsalami F.M., Aigoro N., Mahmoud A.A., Ahmad Z., Haigh P.A., Haas O.C., Rajbhandari S. (2021). Impact of vehicle headlights radiation pattern on dynamic vehicular VLC channel. J. Light. Technol..

[31] Aly B., Elamassie M., Uysal M. Vehicular VLC Channel Model for a Low-Beam Headlight Transmitter. Proceedings of the 2021 17th International Symposium on Wireless Communication Systems (ISWCS).

[32] Aly B., Elamassie M., Eldeeb H.B., Uysal M. Experimental investigation of lens combinations on the performance of vehicular VLC. Proceedings of the 2020 12th International Symposium on Communication Systems, Networks and Digital Signal Processing (CSNDSP).

[33] Amjad M.S., Tebruegge C., Memedi A., Kruse S., Kress C., Scheytt C., Dressler F. An IEEE 802.11 compliant SDR-based system for vehicular visible light communications. Proceedings of the ICC 2019-2019 IEEE International Conference on Communications (ICC).

[34] Ashraf K., Islam S.T., Hosseini A.S., Ashok A. Motion characterization for vehicular visible light communications. Proceedings of the 2019 11th International Conference on Communication Systems & Networks (COMSNETS).

[35] Demir M.S., Eldeeb H.B., Uysal M. (2020). Comp-based dynamic handover for vehicular vlc networks. IEEE Commun. Lett..

[36] Elamassie M., Karbalayghareh M., Miramirkhani F., Kizilirmak R.C., Uysal M. Effect of fog and rain on the performance of vehicular visible light communications. Proceedings of the 2018 IEEE 87th Vehicular Technology Conference (VTC Spring).

[37] Eldeeb H.B., Uysal M. Vehicle-to-vehicle visible light communication: How to select receiver locations for optimal performance?. Proceedings of the 2019 11th International Conference on Electrical and Electronics Engineering (ELECO).

[38] Eldeeb H.B., Sait S.M., Uysal M. (2021). Visible light communication for connected vehicles: How to achieve the omnidirectional coverage?. IEEE Access.

[39] Eldeeb H.B., Miramirkhani F., Uysal M. A path loss model for vehicle-to-vehicle visible light communications. Proceedings of the 2019 15th International Conference on Telecommunications (ConTEL).

[40] Eldeeb H.B., Selmy H.A., Elsayed H.M., Abd El-Samie F.E., Badr R.I. Visible light communication based on CPM-OFDM with chaotic interleaving scheme. Proceedings of the 2017 IEEE Photonics Conference (IPC).

[41] Chaleshtori Z.N., Zvanovec S., Ghassemlooy Z., Eldeeb H.B., Uysal M. (2020). Coverage of a shopping mall with flexible OLED-based visible light communications. Opt. Express.

[42] Islim M.S., Ferreira R.X., He X., Xie E., Videv S., Viola S., Dawson M.D. (2017). Towards 10 Gb/s orthogonal frequency division multiplexing-based visible light communication using a GaN violet micro-LED. Photonics Res..

[43] Mapunda G.A., Ramogomana R., Marata L., Basutli B., Khan A.S., Chuma J.M. (2020). Indoor visible light communication: A tutorial and survey. Wirel. Commun. Mob. Comput..

[44] Modepalli K., Parsa L. (2014). Dual-purpose offline LED driver for illumination and visible light communication. IEEE Trans. Ind. Appl..

[45] Eldeeb H.B., Selmy H.A., Elsayed H.M., Badr R.I. (2018). Interference mitigation and capacity enhancement using constraint field of view ADR in downlink VLC channel. IET Commun..

[46] Kashef M., Abdallah M., Qaraqe K., Haas H., Uysal M. (2015). Coordinated interference management for visible light communication systems. J. Opt. Commun. Netw..

[47] Eldeeb H.B., Selmy H.A., Elsayed H.M., Badr R.I. Co-channel interference cancellation using constraint field of view ADR in VLC channel. Proceedings of the 2017 IEEE Photonics Conference (IPC) Part II.

[48] Lin J.C. (2014). Current activities on exposure limits for humans in the radio-frequency region [Telecommunications health and Safety]. IEEE Antennas Propag. Mag..

[49] Marin-Garcia I., Guerra V., Chavez-Burbano P., Rabadan J., Perez-Jimenez R. (2018). Evaluating the risk of eavesdropping a visible light communication channel. IET Optoelectron..

[50] Zhao S., Xu J., Trescases O. A dimmable LED driver for Visible Light Communication (VLC) based on LLC resonant DC-DC converter operating in burst mode. Proceedings of the 2013 Twenty-Eighth Annual IEEE Applied Power Electronics Conference and Exposition (APEC).

[51] Ma H., Lampe L., Hranilovic S. Integration of indoor visible light and power line communication systems. Proceedings of the 2013 IEEE 17th International Symposium on Power Line Communications and Its Applications.

[52] Guan X., Yang Q., Wang T., Chan C.C.K. (2019). Phase-aligned physical-layer network coding in visible light communications. IEEE Photonics J..

[53] Shao S., Khreishah A., Ayyash M., Rahaim M.B., Elgala H., Jungnickel V., Schulz D., Little T.D.C., Hilt J., Freund R. (2015). Design and analysis of a visible-light-communication enhanced WiFi system. J. Opt. Commun. Netw..

[54] Tao Z., Chen M., Yang Z., Shi J., Zhao J., Liu W. Optimal Control for Digital-Twin THz/VLC Communication Networks. Proceedings of the 2022 IEEE International Conference on Communications Workshops (ICC Workshops).

[55] Eldeeb H.B., Yanmaz E., Uysal M. MAC layer performance of multi-hop vehicular VLC networks with CSMA/CA. Proceedings of the 2020 12th International Symposium On Communication Systems, Networks and Digital Signal Processing (CSNDSP).

[56] Yahia S., Meraihi Y., Ramdane-Cherif A., Gabis A.B., Eldeeb H.B. (2022). Performance evaluation of vehicular Visible Light Communication based on angle-oriented receiver. Comput. Commun..

[57] Gong C., Li S., Gao Q., Xu Z. (2015). Power and rate optimization for visible light communication system with lighting constraints. IEEE Trans. Signal Process..

[58] Mai D.H., Pham A.T. Implementation and evaluation of VLC-based indoor positioning systems for smart supermarkets. Proceedings of the 2018 9th International Conference on Awareness Science and Technology (iCAST).

[59] Aqoob I., Hashem IA T., Mehmood Y., Gani A., Mokhtar S., Guizani S. (2017). Enabling communication technologies for smart cities. IEEE Commun. Mag..

[60] Fekete G., Mészáros G., Udvary E., Fehér G., Berceli T. (2016). Visible light communication channel disturbances and examination of the modulation formats. Int. J. Microw. Wirel. Technol..

[61] Fehér G., Udvary E. (2016). VLC-based indoor localization. Optical Wireless Communications.

[62] Boubakri W., Abdallah W., Boudriga N. A light-based communication architecture for smart city applications. Proceedings of the 2015 17th International Conference on Transparent Optical Networks (ICTON).

[63] Malta L., Miyajima C., Takeda K. (2009). A study of driver behavior under potential threats in vehicle traffic. IEEE Trans. Intell. Transp. Syst..

[64] Carter A. (2005). The status of vehicle-to-vehicle communication as a means of improving crash prevention performance. Tech. Rep..

[65] Meneguette R.I., De Grande R., Loureiro A.A. (2018). Intelligent Transport System in Smart Cities.

[66] DSRC, Toyota Commits Big to. https://www.gardnerweb.com/automotive.

[67] V2X, Cellular. https://www.qualcomm.com/invention/5g/%20cellular-v2x/ecosystem.

[68] Uysal M., Ghassemlooy Z., Bekkali A., Kadri A., Menouar H. (2015). Visible light communication for vehicular networking: Performance study of a V2V system using a measured headlamp beam pattern model. IEEE Veh. Technol. Mag..

[69] Sharda P., Bhatnagar M.R. (2023). Vehicular Visible Light Communication System: Modeling and Visualizing Critical Outdoor Propagation Characteristics. IEEE Trans. Veh. Technol..

[70] Vieira M.A., Vieira M., Louro P., Vieira P. Connected cars: Road-to-vehicle communication through visible light. Proceedings of the Next-Generation Optical Communication: Components, Sub-Systems, and Systems VIII.

[71] Eso E., Ghassemlooy Z., Zvanovec S., Gholami A., Burton A., Hassan N.B., Younus O.I. Experimental demonstration of vehicle to road side infrastructure visible light communications. Proceedings of the 2019 2nd West Asian Colloquium on Optical Wireless Communications (WACOWC).

[72] Al-Kinani A., Sun J., Wang C.X., Zhang W., Ge X., Haas H. (2018). A 2-D non-stationary GBSM for vehicular visible light communication channels. IEEE Trans. Wirel. Commun..

[73] Eldeeb H.B., Eso E., Jarchlo E.A., Zvanovec S., Uysal M., Ghassemlooy Z., Sathian J. (2021). Vehicular VLC: A ray tracing study based on measured radiation patterns of commercial taillights. IEEE Photonics Technol. Lett..

[74] Eldeeb H.B., Elamassie M., Uysal M. Vehicle-to-infrastructure visible light communications: Channel modelling and capacity calculations. Proceedings of the 2020 12th International Symposium on Communication Systems, Networks and Digital Signal Processing (CSNDSP).

[75] Eso E., Burton A., Hassan N.B., Abadi M.M., Ghassemlooy Z., Zvanovec S. Experimental investigation of the effects of fog on optical camera-based VLC for a vehicular environment. Proceedings of the 2019 15th International Conference on Telecommunications (ConTEL).

[76] Karbalayghareh M., Miramirkhani F., Eldeeb H.B., Kizilirmak R.C., Sait S.M., Uysal M. (2020). Channel modelling and performance limits of vehicular visible light communication systems. IEEE Trans. Veh. Technol..

[77] Yahia S., Meraihi Y., Ramdane-Cherif A., Ho T.D., Eldeeb H.B. (2023). Enhancement of Vehicular Visible Light Communication Using Spherical Detector and Custom Lens Combinations. IEEE Access.

[78] Yahia S., Meraihi Y., Refas S., Gabis A.B., Ramdane-Cherif A., Eldeeb H.B. (2022). Performance study and analysis of MIMO visible light communication-based V2V systems. Opt. Quantum Electron..

[79] Aghaei F., Eldeeb H.B., Uysal M. (2023). A comparative evaluation of propagation characteristics of vehicular VLC and MMW channels. IEEE Trans. Veh. Technol..

[80] Seminara M., Nawaz T., Caputo S., Mucchi L., Catani J. (2020). Characterization of field of view in visible light communication systems for intelligent transportation systems. IEEE Photonics J..

[81] Caputo S., Mucchi L., Cataliotti F., Seminara M., Nawaz T., Catani J. (2021). Measurement-based VLC channel characterization for I2V communications in a real urban scenario. Veh. Commun..

[82] Arafa N.A., Arafa M.S., Abd El-atty S.M., El-Samie FE A., Eldeeb H.B. (2023). Capacity analysis of infrastructure-to-vehicle visible light communication with an optimized non-orthogonal multiple access scheme. Opt. Quantum Electron..

[83] Eldeeb H.B., Elamassie M., Sait S.M., Uysal M. (2022). Infrastructure-to-Vehicle Visible Light Communications: Channel Modelling and Performance Analysis. IEEE Trans. Veh. Technol..

[84] Zadobrischi E. System prototype proposed for vehicle communications based on VLC-RF technologies adaptable on infrastructure. Proceedings of the 2020 International Conference on Development and Application Systems (DAS).

[85] Nawaz T., Seminara M., Caputo S., Mucchi L., Catani J. (2020). Low-latency VLC system with Fresnel receiver for I2V ITS applications. J. Sens. Actuator Netw..

[86] Avătămăniței S.A., Beguni C., Căilean A.M., Dimian M., Popa V. (2021). Evaluation of misalignment effect in vehicle-to-vehicle visible light communications: Experimental demonstration of a 75 meters link. Sensors.

[87] Tettey D.K., Elamassie M., Uysal M. Experimental Investigation of Angle Diversity Receiver for Vehicular VLC. Proceedings of the 29th Annual International Conference on Mobile Computing and Networking.

[88] Dimitrov S., Haas H. (2015). Principles of LED Light Communications: Towards Networked Li-Fi.

[89] Kruse S., Kress C., Memedi A., Tebruegge C., Amjad M.S., Scheytt C., Dressler F. Design of an automotive visible light communications link using an off-the-shelf led headlight. Proceedings of the ANALOG 2018; 16th GMM/ITG-Symposium.

[90] Tian Z., Wright K., Zhou X. The darklight rises: Visible light communication in the dark. Proceedings of the 22nd Annual International Conference on Mobile Computing and Networking.

[91] Class T., Memedi A., Dressler F. Reduced multiuser-interference for vehicular VLC using SDMA and matrix headlights. Proceedings of the 2019 IEEE Global Communications Conference (GLOBECOM).

[92] Class T., Zhang Q., Falko D. Optical interference reduction with spatial filtering receiver for vehicular visible light communication. Proceedings of the 2019 IEEE Intelligent Transportation Systems Conference (ITSC).

[93] Khalighi M.A., Uysal M. (2014). Survey on free space optical communication: A communication theory perspective. IEEE Commun. Surv. Tutor..

[94] Jiang R., Wang Q., Haas H., Wang Z. (2017). Joint user association and power allocation for cell-free visible light communication networks. IEEE J. Sel. Areas Commun..

[95] Luo P., Ghassemlooy Z., Le Minh H., Bentley E., Burton A., Tang X. (2015). Performance analysis of a car-to-car visible light communication system. Appl. Opt..

[96] Claas T., Memedi A., Dressler F. Empirical characterization of the NLOS component for vehicular visible light communication. Proceedings of the 2019 IEEE Vehicular Networking Conference (VNC).

[97] Turan B., Narmanlioglu O., Koc O.N., Kar E., Coleri S., Uysal M. (2022). Measurement based non-line-of-sight vehicular visible light communication channel characterization. IEEE Trans. Veh. Technol..

[98] Alsalami F.M., Haas O.C., Al-Kinani A., Wang C.X., Ahmad Z., Rajbhandari S. (2021). Impact of dynamic traffic on vehicle-to-vehicle visible light communication systems. IEEE Syst. J..

[99] Cui K. (2012). Physical Layer Characteristics and Techniques for Visible Light Communications.

[100] Turan B., Gurbilek G., Uyrus A., Ergen S.C. Vehicular VLC frequency domain channel sounding and characterization. Proceedings of the 2018 IEEE Vehicular Networking Conference (VNC).

[101] Yoo J.H., Jang J.S., Kwon J.K., Kim H.C., Song D.W., Jung S.Y. (2016). Demonstration of vehicular visible light communication based on LED headlamp. Int. J. Automot. Technol..

[102] Stepniak G., Schüppert M., Bunge C.A. (2015). Advanced modulation formats in phosphorous LED VLC links and the impact of blue filtering. J. Light. Technol..

[103] Singh G., Srivastava A., Bohara V.A. On feasibility of vlc based car-to-car communication under solar irradiance and fog conditions. Proceedings of the 1st International Workshop on Communication and Computing in Connected Vehicles and Platooning.

[104] Singh G., Srivastava A., Bohara V.A. Impact of Weather Conditions and Interference on the Performance of VLC based V2V Communication. Proceedings of the 2019 21st International Conference on Transparent Optical Networks (ICTON).

[105] Danys L., Martinek R., Slanina Z., Kolarik J., Jaros R. The impact of rain on performance of visible light communication. Proceedings of the Photonics Applications in Astronomy, Communications, Industry, and High-Energy Physics Experiments 2019.

[106] Georlette V., Bette S., Brohez S., Pérez-Jiménez R., Point N., Moeyaert V. (2020). Outdoor visible light communication channel modeling under smoke conditions and analogy with fog conditions. Optics.

[107] Joshi K., Roy N., Singh G., Bohara V.A., Srivastava A. Experimental observations on the feasibility of VLC-based V2X communications under various environmental deterrents. Proceedings of the 2019 IEEE International Conference on Advanced Networks and Telecommunications Systems (ANTS).

[108] Cui Z., Yue P., Yi X., Li J. Research on non-uniform dynamic vehicle-mounted VLC with receiver spatial and angular diversity. Proceedings of the ICC 2019-2019 IEEE International Conference on Communications (ICC).

[109] Morales-Céspedes M., Quidan A.A., Armada A.G. Experimental evaluation of the reconfigurable photodetector for blind interference alignment in visible light communications. Proceedings of the 2019 27th European Signal Processing Conference (EUSIPCO).

[110] Nawaz T., Seminara M., Caputo S., Mucchi L., Cataliotti F.S., Catani J. (2019). IEEE 802.15. 7-compliant ultra-low latency relaying VLC system for safety-critical ITS. IEEE Trans. Veh. Technol..

[111] You X., Zhong Y., Chen J., Yu C. (2020). Mobile channel estimation based on decision feedback in vehicle-to-infrastructure visible light communication systems. Opt. Commun..

[112] Eldeeb H.B., Eso E., Uysal M., Ghassemlooy Z., Zvanovec S., Sathian J. Vehicular visible light communications The impact of taillight radiation pattern. Proceedings of the 2020 IEEE Photonics Conference (IPC).

[113] Al-Kinani A., Wang C.X., Zhu Q., Fu Y., Aggoune EH M., Talib A., Al-Hasaani N.A. (2020). A 3D non-stationary GBSM for vehicular visible light communication MISO channels. IEEE Access.

[114] Memedi A., Tebruegge C., Jahneke J., Dressler F. Impact of vehicle type and headlight characteristics on vehicular VLC performance. Proceedings of the 2018 IEEE Vehicular Networking Conference (VNC).

[115] Vieira M.A., Vieira M., Louro P., Vieira P. (2020). Redesign of the trajectory within a complex intersection for visible light communication ready connected cars. Opt. Eng..

[116] Singh G., Srivastava A., Bohara V.A., Liu Z., Noor-A-Rahim M., Ghatak G. (2022). Heterogeneous visible light and radio communication for improving safety message dissemination at road intersection. IEEE Trans. Intell. Transp. Syst..

[117] Vieira M.A., Vieira M., Louro P., Vieira P. Vehicular visible light communication in a traffic controlled intersection. Proceedings of the Optical Sensors 2021.

[118] Abualhoul M.Y., Marouf M., Shagdar O., Nashashibi F. Platooning control using visible light communications: A feasibility study. Proceedings of the 16th International IEEE Conference on Intelligent Transportation Systems (ITSC 2013).

[119] Schettler M., Memedi A., Dressler F. Deeply integrating visible light and radio communication for ultra-high reliable platooning. Proceedings of the 2019 15th Annual Conference on Wireless On-demand Network Systems and Services (WONS).

[120] Abualhoul M.Y., Marouf M., Shag O., Nashashibi F. Enhancing the field of view limitation of visible light communication-based platoon. Proceedings of the 2014 IEEE 6th International Symposium on Wireless Vehicular Communications (WiVeC 2014).

[121] Béchadergue B., Chassagne L., Guan H. Suitability of visible light communication for platooning applications: An experimental study. Proceedings of the 2018 Global LIFI Congress (GLC).

[122] Tseng H.Y., Wei Y.L., Chen A.L., Wu H.P., Hsu H., Tsai H.M. Characterizing link asymmetry in vehicle-to-vehicle visible light communications. Proceedings of the 2015 IEEE Vehicular Networking Conference (VNC).

[123] Memedi A., Tsai H.M., Dressler F. Impact of realistic light radiation pattern on vehicular visible light communication. Proceedings of the GLOBECOM 2017-2017 IEEE Global Communications Conference.

[124] Sharda P., Reddy G.S., Bhatnagar M.R., Ghassemlooy Z. (2022). A Comprehensive Modeling of Vehicle-To-Vehicle Based VLC System under Practical Considerations, an Investigation of Performance, and Diversity Property. IEEE Trans. Commun..

[125] Al-Kinani A., Wang C.X., Zhou L., Zhang W. (2018). Optical wireless communication channel measurements and models. IEEE Commun. Surv. Tutor..

[126] Mie G. (1908). Beiträge zur Optik trüber Medien, speziell kolloidaler Metallösungen. Ann. Der Phys..

[127] Kim I.I., McArthur B., Korevaar E.J. Comparison of laser beam propagation at 785 nm and 1550 nm in fog and haze for optical wireless communications. Proceedings of the Optical Wireless Communications III.

[128] Ghassemlooy Z., Popoola W., Rajbhandari S. (2019). Optical Wireless Communications: System and Channel Modelling with Matlab^®^.

[129] Mohammed N.A., El-Wakeel A.S., Malekmohammadi A., Aly M.H. (2012). Performance evaluation of FSO link under NRZ-RZ line codes, different weather conditions and receiver types in the presence of pointing errors. Open Electr. Electron. Eng. J..

[130] Yu S.H., Shih O., Tsai H.M., Wisitpongphan N., Roberts R. (2013). Smart automotive lighting for vehicle safety. IEEE Commun. Mag..

[131] Yamazato T., Takai I., Okada H., Fujii T., Yendo T., Arai S., Kawahito S. (2014). Image-sensor-based visible light communication for automotive applications. IEEE Commun. Mag..

[132] Goto Y., Takai I., Yamazato T., Okada H., Fujii T., Kawahito S., Arai S., Yendo T., Kamakura K. (2016). A new automotive VLC system using optical communication image sensor. IEEE Photonics J..

[133] Chen A.L., Wu H.P., Wei Y.L., Tsai H.M. Time variation in vehicle-to-vehicle visible light communication channels. Proceedings of the 2016 IEEE Vehicular Networking Conference (VNC).

[134] Eldeeb H.B., Elamassie M., Uysal M. Performance analysis and optimization of cascaded i2v and v2v vlc links. Proceedings of the 2021 17th International Symposium on Wireless Communication Systems (ISWCS).

[135] Cailean A.-M., Cagneau B., Chassagne L., Topsu S., Alayli Y., Dimian M. Visible light communications cooperative architecture for the intelligent transportation system. Proceedings of the 2013 IEEE 20th Symposium on Communications and Vehicular Technology in the Benelux (SCVT).

[136] Refas S., Acheli D., Yahia S., Meraihi Y., Ramdane-Cherif A., Gabis A.B., Eldeeb H.B. Performance Analysis of Multi-Hop V2V VLC System under Atmospheric Weather Conditions. Proceedings of the 2022 IEEE 9th International Conference on Sciences of Electronics, Technologies of Information and Telecommunications (SETIT).

[137] Demir M.S., Eldeeb H., Uysal M. (2022). Relay-assisted handover technique for vehicular VLC networks. ITU J. Future Evol. Technol..

[138] Zhan L., Zhao H., Zhang W., Lin J., Zhao X. (2022). Performance analysis and node selection of intelligent reflecting surface-aided visible light communication for parallel vehicles. Wireless Commun. Mobile Comput..

[139] Singh G., Srivastava A., Bohara V.A. (2022). Visible light and reconfigurable intelligent surfaces for beyond 5G V2X communication networks at road intersections. IEEE Trans. Veh. Technol..

[140] Zhan L., Zhao H., Zhang W., Lin J. (2022). An optimal scheme for the number of mirrors in vehicular visible light communication via mirror array-based intelligent reflecting surfaces. Photonics.

[141] Eldeeb H.B., Naser S., Bariah L., Muhaidat S. (2023). Energy and Spectral Efficiency Analysis for RIS-Aided V2V-Visible Light Communication. IEEE Commun. Lett..

[142] Sipani J., Sharda P., Bhatnagar M.R. (2023). Modeling and Design of IRS-Assisted FSO System Under Random Misalignment. IEEE Photonics J..

[143] Fan B., Wu Y., He Z., Chen Y., Quek T.Q., Xu C.-Z. (2021). Digital twin empowered mobile edge computing for intelligent vehicular lane-changing. IEEE Netw..

[144] Wang Z., Gupta R., Han K., Wang H., Ganlath A., Ammar N., Tiwari P. (2022). Mobility digital twin: Concept, architecture, case study, and future challenges. IEEE Internet Things J..

[145] Ding C., Ho I.W.-H. (2022). Digital-twin-enabled city-model-aware deep learning for dynamic channel estimation in urban vehicular environments. IEEE Trans. Green Commun. Netw..

[146] Wang Z., Han K., Tiwari P. (2022). Digital twin-assisted cooperative driving at non-signalized intersections. IEEE Trans. Intell. Veh..

[147] Gong Y., Wei Y., Feng Z., Yu F.R., Zhang Y. (2023). Resource allocation for integrated sensing and communication in digital twin enabled internet of vehicles. IEEE Trans. Veh. Technol..

[148] Eldeeb H., Naser S., Bariah L., Muhaidat S., Uysal M. (2023). Digital Twin-Assisted OWC: Towards Smart and Autonomous 6G Networks. Authorea Prepr..

[149] Aboagye S., Ndjiongue A.R., Ngatched T.M., Dobre O.A., Poor H.V. (2022). RIS-assisted visible light communication systems: A tutorial. IEEE Commun. Surv. Tutor..

[150] Sharma T., Chehri A., Fortier P. (2021). Reconfigurable intelligent surfaces for 5G and beyond wireless communications: A comprehensive survey. Energies.

